# *HISET*: Hybrid interpretable strategies with ensemble techniques for respiratory sound classification

**DOI:** 10.1016/j.heliyon.2023.e18466

**Published:** 2023-07-22

**Authors:** Sunil Kumar Prabhakar, Dong-Ok Won

**Affiliations:** Department of Artificial Intelligence Convergence, Hallym University, Chuncheon, Gangwon-do, South Korea

**Keywords:** Respiratory sound classification, Feature extraction, Feature selection, Classification

## Abstract

The human respiratory systems can be affected by several diseases and it is associated with distinctive sounds. For advanced biomedical signal processing, one of the most complex issues is automated respiratory sound classification. In this research, five Hybrid Interpretable Strategies with Ensemble Techniques (HISET) which are quite interesting and robust are proposed for the purpose of respiratory sounds classification. The first approach is termed as an Ensemble GSSR technique which utilizes $L_{2}$ Granger Analysis and the proposed Supportive Ensemble Empirical Mode Decomposition (SEEMD) technique and then Support Vector Machine based Recursive Feature Elimination (SVM-RFE) is used for feature selection and followed by classification with Machine Learning (ML) classifiers. The second approach proposed is the implementation of a novel Realm Revamping Sparse Representation Classification (RR-SRC) technique and third approach proposed is a Distance Metric dependent Variational Mode Decomposition (DM-VMD) with Extreme Learning Machine (ELM) classification process. The fourth approach proposed is with the usage of Harris Hawks Optimization (HHO) with a Scaling Factor based Pliable Differential Evolution (SFPDE) algorithm termed as HHO-SFPDE and it is classified with ML classifiers. The fifth or the final approach proposed analyzes the application of dimensionality reduction techniques with the proposed Gray Wolf Optimization based Support Vector Classification (GWO-SVC) and another parallel approach utilizes a similar kind of analysis with the Grasshopper Optimization Algorithm (GOA) based Sparse Autoencoder. The results are examined for ICBHI dataset and the best results are shown for the 2-class classification when the analysis is carried out with Manhattan distance-based VMD-ELM reporting an accuracy of 95.39%, and for 3-class classification Euclidean distance-based VMD-ELM reported an accuracy of 90.61% and for 4-class classification, Manhattan distance-based VMD-ELM reported an accuracy of 89.27%.

## 1 Introduction

In recent decades, the automated examination of respiratory sounds has triggered a substantial interest to researchers [1]. Detecting the deformities in the early phases of respiratory problems is done by the automated classification of respiratory sounds. A vital indicator of respiratory related disorders is examined by this respiratory sound. Factors such as air gesticulation, changes in the lung tissue and lung secretions have a great influence on the sound emitted by a person during respiration. With the help of digital stethoscopes and other recording schemes, the recording of these sounds can be done easily [2]. This digital data aids the way for ML experts to automatically diagnose different respiratory disorders such as bronchitis, pneumonia, asthma etc. When normal and advanced computational techniques are performed for getting a deeper insight of these sounds, there is a huge improvement in the detection and classification of respiratory diseases [3]. With the help of various tools like sonograph and stethoscope, the respiratory related checkups are done for research purposes and the audio samples can be easily collected from the individuals. To identify the pathology with respect to this audio, it requires a huge amount of time and effort. The diagnosis of any disease is related to the visual or audio information procured from various techniques. As it is quite difficult to find a medical specialist who has a high skill and experience, automated techniques have come into existence [4]. As far as respiratory related disease is concerned, an audio sample considered from the sonograph or stethoscope from different fragments of the body seems to be useful for further analysis. Different hearing sounds such as normal, wheeze, crackle etc. can be analyzed and can aid in the recognition of the disease and so it is evident that the audio samples help in the classification and analysis of respiratory diseases.

Artificial Intelligence (AI) assisted auscultation helps to easily recognize the sound patterns and therefore abnormalities could be easily identified [5]. Due to the airflow in the respiratory region, the respiratory sounds are produced and it is split into two categories such as normal or abnormal sound [6]. When there is no pulmonary disorder, a normal respiratory sound is produced and it consists of bronchial, tracheal, and vesicular sounds. If there is any disease in the lungs or bronchitis, abnormal respiratory sounds are caused. Depending on the characteristics, the location and the mechanism of production, the respiratory sounds can be further examined and analyzed. In order to conquer the subjectivity of human auscultation, a standardized and automated system is required to analyze the respiratory sounds accurately [7]. ML techniques and deep learning techniques are widely used for analysis in the classification of respiratory disorders, Electroencephalography (EEG), chest radiograph etc. [8]. New approaches are always developed by these AI techniques for an exact and accurate analysis of respiratory sounds. There are two important parts of respiratory sound analysis in the ML perspective. The first one lies in developing predictive models dependent on well-known ML techniques such as Support Vector Machine (SVM), Decision Trees (DT), Random Forest (RF), Artificial Neural Networks (ANN), K-nearest neighbor (KNN), Naïve Bayesian Classifier (NBC) and deep learning techniques such as Long Short-Term Memory (LSTM), Bi-directional LSTM (BiLSTM), Convolutional Neural Network (CNN), Gated Recurrent Unit (GRU) and Bi-directional GRU (BiGRU) [9]. The second one lies in defining the most appropriate features expressing the characteristics of respiratory sound and then extracting it using Short Time Fourier Transform (STFT), Mel-Frequency Cepstral Coefficients (MFCC), Singular Spectrum Analysis (SSA), Signal Augmentation (SA) [9] etc.

Some of the prominent works for the respiratory sound classification are discussed as follows. The pulmonary diseases were recognized from lung sounds using CNN and LSTM for 213 subjects and the number of classes used here are 6 (normal, asthma, pneumonia, bronchiectasis, Chronic Obstructive Pulmonary Disease (COPD), and heart failure) and the best accuracy of 99.62% was obtained using hybrid CNN-BiLSTM [10]. For breath phase and adventitious sound detection, eight Recurrent Neural Networks (RNN) were utilized for 279 subjects and the number of classes used here are 6 (wheeze, inhalation, rhonchus, stridor, exhalation, crackles) and the best F1-score of 80.6% was obtained when CNN-BiGRU was implemented [11]. The amalgamation of STFT and MFCC features were done and the classification of lung sounds using Depthwise separable CNN (DS-CNN) models was implemented for 12,691 recordings and the number of classes used here were 4 (normal, wheeze, crackle, unknown) and the best accuracy of 85.74% was obtained [12]. The wavelet coefficients and ML techniques were used for sensing the respiratory sounds for 130 subjects and the number of classes used here are 3 (crackles, rhonchi, normal) and a high classification accuracy of 85.43% was procured when classified with ANN [13]. The feature extraction techniques for pulmonary sound investigation based on Empirical Mode Decomposition (EMD) for 30 subjects using ML classifiers was done and a high classification accuracy of 94.16% was achieved when EMD was classified with ANN [14]. The detection of pathological breath sounds in children using AI techniques for 25 subjects was done and the number of classes classified here are 2 (crackles and wheeze) and the metrics analyzed here are quite different from the conventional ones such as Positive Percent Agreement (PPA) and Negative Percent Agreement (NPA) [15]. The deep learning was applied to classify the extremity of COPD for 120 recordings and the number of classes classified here are 2 classes (crackles and wheeze) and a high classification accuracy of 95.84% was reaped with Deep Belief Network (DBN) [16]. The semi-supervised deep investigation techniques were applied to the lung sound investigation for 284 subjects and 11627 recordings and the number of classes classified here are 2 (crackles and wheeze) and a high Area under the ROC curve (AUC) of 0.86 was obtained with SVM [17]. The feature evaluation strategy for classification of wheezes and normal respiratory sounds with linear classifiers was studied by Pramono et al. [18] and another interesting strategy proposed in the recent literature is detection of COVID-19 from the respiratory sound recordings with the help of transformers which reported an AUC over 94% [19]. An efficient feature stacking methodology with a dense lightweight CNN-BiGRU skip connections was proposed for the respiratory sound classification reporting an accuracy of 92.3% [20]. An idea dependent on a Euclidean distance method along with Maximum Relevancy Minimum Redundancy (MRMR) feature selection technique and KNN classifier, a classification accuracy of 91.02% was achieved for respiratory sound classification [21] and a deep learning CNN was utilized to designate 1918 respiratory sounds (rhonchi, normal, wheezes, crackles) reporting a classification accuracy of 85.7% [22].

Some of the old publicly available respiratory sound databases are *Secrets Heart & Lung Sounds Workshops database*, *Understanding Heart Sounds and Murmurs* database, R.A.L.E. repository database, East Tennessee State University repository database, *Fundamentals of Lung and Heart Sounds* database, *Auscultation Skills: Breath and Heart Sounds-*fourth edition database, Heart and lung sounds reference library database, Littmann repository database, SoundCloud repository database and Kraman Lung Sound dataset [23]. The most recently published dataset which is quite famously used in recent times is the International Conference on Biomedical and Health Informatics (ICBHI) dataset [23]. The following details reports the prominent previous work specifically dealt with ICBHI dataset. For a two-class classification problem in ICBHI dataset, ResNet 34 was utilized and a F-score of 77% was obtained [24], Deep Neural Networks (DNN) was utilized reporting an accuracy of 82% [25], CNN was utilized reporting an accuracy of 83.7% [26] and Multilayer Perceptron (MLP) was utilized reporting an accuracy of 99.2% [27]. When a 3-class classification problem in ICBHI dataset was analyzed, a lightweight CNN model was proposed reporting a classification accuracy of 98.9% [28] and the results with simple ML classifiers reported an accuracy of 97.7% [29], Piccolo pattern-based analysis reported 99.31% [30], and hybrid of MFCC, Mel spectrogram, ChromCENS with CNN reported a F-score of 0.99 [31]. A lot of researchers have reported results with 4-class classification problem in the ICBHI dataset such as the analysis with STFT and wavelets with Bi-ResNet architecture producing a classification accuracy of 50.16% [32], a hybrid CNN-LSTM with Focal loss function reporting an accuracy of 76.39% [33], a directed acyclic graph with Hidden Markov Model (DAG-HMM) produced an accuracy of 50.1% [34], CNN reported an accuracy of 50% [35], a hybrid CNN-RNN architecture produced an accuracy of 71.81% [36], CNN with autoencoders together produced a classification accuracy of 49% [37], an inception based neural network with multi spectrogram ensembles generated a classification accuracy of 86% [38], CNN was utilized reporting an accuracy of 83.7% [26] and Bi-ResNet deep learning algorithm induced a classification accuracy of 52.79% [39]. The CNN with attention mechanism was utilized by Li et al. and it assembled a classification accuracy of 67% for the 5-class classification problem [40]. For a 6-class classification problem, ensemble classifiers such as Boosted Decision trees was utilized reporting a classification accuracy of 98.27% [41], 2D-CNN reporting an accuracy of 92% [42] and MFCC with CNN reported an accuracy of 90.21% [43], and a Deep Neural Network (DNN) model was analyzed where a classification accuracy of 95.67% was obtained [44]. A 7-class classification problem was dealt with the application of Piccolo pattern with KNN classifier reporting a very high classification accuracy of 99.45% [30], and an 8-class classification problem was also dealt with the same Piccolo pattern and KNN classifier reporting a very high accuracy of 99.19% [30]. Generally, for signal processing applications, a lot of generalized works are incorporated for concepts such as artificial intelligence based robust hybrid algorithms [45], entropy-based analysis [46], swarm intelligence models [47], chaotic salp swarm algorithm [48], chaotic-based interactive autodidactic school algorithm [49], slime mold algorithm [50], quantum-inspired algorithm [51], improved cuckoo-search algorithm [52], gorilla troop optimization algorithm [53] and sparrow search optimization algorithm [54].

The major benefactions of this work are as follows:

a) The pre-processing in this work was done using the standard Independent Component Analysis (ICA) technique. Initially a hybrid approach called Ensemble GSSR technique is proposed which utilizes $L_{2}$ Granger Analysis and the proposed Supportive Ensemble Empirical Mode Decomposition (SEEMD) technique and the components derived from it is merged. Then SVM-RFE is used for feature selection and followed by classification with ML classifiers.
b) The second approach proposed is the implementation of a novel Realm Revamping Sparse Representation Classification (RR-SRC). The local information conserved in RR- SRC is analyzed and then the RR-SRC model is built and analyzed.
c) The third approach proposed is a Distance Metric dependent Variational Mode Decomposition (DM-VMD) with Extreme Learning Machine (ELM) classification process. Various distance metrics were analyzed with VMD such as Euclidean distance, Manhattan distance, Chebyshev distance, Jaccard Index and Sorensen-Dice Index.
d) The fourth approach proposed is done with the merging of Harris Hawks Optimization (HHO) with a proposed Scaling Factor based Pliable Differential Evolution (SFPDE) algorithm termed together as HHO-SFPDE technique and analyzed with ML classifiers.
e) The final approach proposed analyzes the application of dimensionality reduction techniques with the proposed Gray Wolf Optimization based Support Vector Classification (GWO-SVC) and another parallel approach utilizes the analysis with the Grasshopper Optimization Algorithm (GOA) with Sparse Autoencoder.

**Fig. 1:**
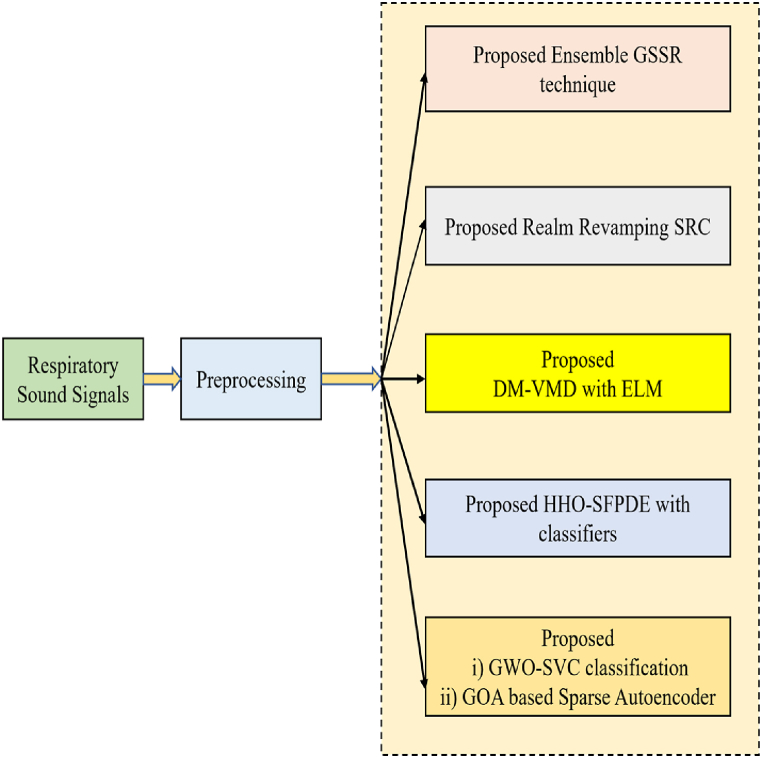
Simplified elucidation of the work.

The overall and simplified drawing of the work is represented in Fig. 1.

The organization of the paper is as follows. Section 2 describes the first proposed technique Ensemble GSSR method. Section 3 describes the second proposed technique RR-SRC model. Section 4 expresses the third proposed technique Hybrid Distance Metric Based VMD with ELM. Section 5 explains about the proposed HHO-SFPDE based Classification. Section 6 describes the proposed Dimensionality Reduction Techniques with Metaheuristic based Support Vector Classification and Sparse Autoencoders. The results and discussion are expressed in section 7 and the conclusion is expressed in section 8.

## 2 Proposed technique 1: ensemble GSSR method

**Fig. 2:**
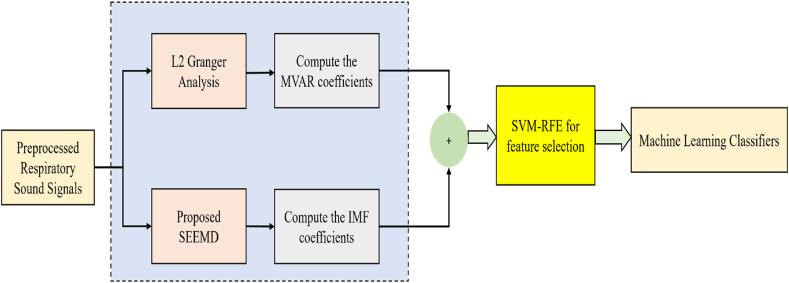
Proposed ensemble GSSR method.

In this proposed Ensemble GSSR technique, the Multivariate AutoRegressive Model (MVAR) components are obtained by means of using $L_{2}$ Granger Analysis and then the IMF components are obtained from the proposed SEEMD technique. The Ensemble of these two components are done and then using the SVM-RFE technique, the essential features are selected and finally classified with the ML classifiers. Fig. 2 expresses the overall model of the proposed Ensemble GSSR method.

### 2.1 $L_{2}$ Granger Analysis

Depending on a Multivariate AutoRegressive Model (MVAR), Granger analysis is usually implemented [55]. To assess the unique relationship, present between the multiple sets of time series data, $L_{2}$ Granger Analysis is used. The MVAR parameters are generally computed with good accuracy so that the reliability of the final relationship is assessed, thereby having a profound influence on the accuracy of the entire Granger correlation network. To estimate the parameters of the MVAR models, there are multiple strategies. Assuming that there are p stationary stochastic models with $Z_{i}\left(t\right)\in R$ time domain observations such that i=1,2,...,p;t=1,2,...,T. The influence of the activity of $Z_{i}\left(t\right)$ on $Z_{j}\left(t\right)$ is defined by the vector of coefficients denoted as $c_{ij}\left(i=1,2,...,p;j=1,2,...,p\right)$. Between the expected $Z_{k}$ and the predicted ${\hat{Z}}_{k}$, the variance of the residuals is expressed as $\sum_{k}\left(k=1,2,...,p\right)$.

Assuming that $X_{k}={\left[c_{1k}\left(l\right),...,c_{1k}\left(l\right),...,c_{pk}\left(l\right),...,c_{pk}\left(l\right)\right]}^{T}$ are the total number of multivariate autoregressive coefficients, with 'p' denoting the time series once again and $y_{k}=\left[z_{k}\left(l+1\right),z_{k}\left(l+2\right),...,z_{k}\left(n\right)\right]$ representing the n−l elements to be predicted for $z_{k}$, where the length of the signal is indicated by n. The degree matrix $C\in R^{\left(n-l\right)\times \left(p\times l\right)}$ is expressed as:

(1) $$C=\left[Q_{1},Q_{2},...,Q_{k},...,Q_{p}\right]$$

In such a situation;

(2) $$Q_{i}=\left[\begin{array}{cccc} z_{i}\left(l\right) & z_{i}\left(l-1\right) & .. & z_{i}\left(1\right)\\ z_{i}\left(l+1\right) & z_{i}\left(l\right) & .. & z_{i}\left(2\right)\\ : & : & : & :\\ z_{i}\left(n-1\right) & z_{i}\left(n-2\right) & .. & z_{i}\left(n-l\right) \end{array}\right]$$

The objective term utilized in the $L_{2}$ norm space is expressed as:

(3) $$\underset{x_{k}}{\mathrm{argmin}}f_{k}\left(X_{k}\right)={\left\|y_{k}-CX_{k}\right\|}_{2}^{2}$$

The $L_{2}$ norm of the vector is expressed as ‖·‖ and the objective function $f_{k}\left(X_{k}\right)$ which is minimized by the best solution is expressed as “argmin”. The following formulation is obtained if the derivate of equation (3) is considered with references to $X_{k}$ and the condition (dfkdXk)=0 and is represented as:

(4) $$2C^{T}CX_{k}-2C^{T}y_{k}=0$$

For the process $z_{k}$, the MVAR coefficient is expressed as:

(5) $$X_{k}=\left\{\begin{array}{c} {\left(C^{T}C\right)}^{-1}C^{T}y_{k}\begin{array}{cc} & if\begin{array}{cc} C^{T}C & is\begin{array}{cc} & non-\sin\;gular \end{array} \end{array} \end{array}\\ \left(C^{T}C\right)+C^{T}y_{k}\begin{array}{cc} & if\begin{array}{cc} & C^{T}C\begin{array}{cc} & is\begin{array}{cc} & \sin\;gular \end{array} \end{array} \end{array} \end{array} \end{array}\right\}$$

### 2.2 Supportive ensemble EMD

One of the famous improved signal decomposition procedures for Ensemble Empirical Mode Decomposition (EEMD) [56] is proposed here as Supportive Ensemble Empirical Mode Decomposition (SEEMD). The modal aliasing in the EMD decomposition technique is suppressed by this method. The complex processing in EEMD and the issue of residual white noise is solved by the proposed SEEMD. The decomposition activity of the SEEMD technique is dependent on the EMD as a couple of subsidiary white noise with the matching amplitude and converse sign to the respiratory sound signals is added. The decomposition of these respiratory signals into many intrinsic mode functions (IMFs) and residuals with a deep and clear inference makes it easy to proceed. The remaining aggregation of noise in the reconstruction information will mitigate as the total number of appended noises escalates and ultimately the final residual consignment is ignored completely. The decomposition procedure of the SEEMD is considered as follows:

Step 1 Initially, to form the two novel decomposition signals, a couple of Gaussian white noises which is random in nature with matching amplitude and converse signs are annexed to the respiratory sound signals as follows:

(6) $$\left\{ \begin{array}{c} S_{i+}\left(t\right)=S\left(t\right)+N_{i}^{+}\left(t\right)\\ S_{-i}\left(t\right)=S\left(t\right)+N_{i}^{-}\left(t\right) \end{array}\right.$$

where S(t) is the sound signal. The white noise appended for the 'i' time is expressed as $N_{i}\left(t\right)$. The signal procured by means of appending the positive white noise for the 'i' time is represented as $S_{+i}\left(t\right)$. The signal derived by means of prepending the negative white noise for 'i' time is represented as $S_{-i}\left(t\right)$. The value is usually in the range of 0.02–0.4 times the Standard Deviation of the indigenous signal.

Step 2 In order to acquire the IMF constituents and the residual denominations, the decomposition of $S_{+i}\left(t\right)$ and $S_{-i}\left(t\right)$ are done by the EMD algorithm.

(7) $$S_{+i}\left(t\right)=\sum_{j=1}^{q}I_{+ij}\left(t\right)+R_{+i}\left(t\right)$$

(8) $$S_{-i}\left(t\right)=\sum_{j=1}^{q}I_{-ij}\left(t\right)+R_{-i}\left(t\right)$$

where the j IMF constituent from $S_{+i}\left(t\right)$ decomposition is indicated by $I_{+ij}\left(t\right)$ . The j IMF constituent from $S_{-i}\left(t\right)$ decomposition in indicated by $I_{-ij}\left(t\right)$. The corresponding residual term is reported by $R_{+i}\left(t\right)$ and $R_{-i}\left(t\right)$ respectively.

Step 3 For 'q' times, the step 1 and step 2 are replicated, and unless the decomposition of the residual expressions can no longer be done, the addition of the random white noise is done.
Step 4 After the decomposition, the mean value of the IMF constituents secured are calculated and then the mean value is taken as the ultimate outcome of the IMF constituent.

(9) $$C_{j}\left(t\right)=\frac{1}{2q}\sum_{i=1}^{q}\left(I_{+ij}\left(t\right)+I_{-ij}\left(t\right)\right)$$

where the initial IMF component attained by SEEMD is indicated by $C_{j}\left(t\right)$.

### 2.3 SVM-RFE

Once the MVAR coefficients and the IMF components are calculated, it is ensembled together and then it is progressed to feature selection stage. One of the famous feature selection technique dependent on feature sorting technology is SVM-RFE [57]. With the help of greedy strategy, the features are ranked by RFE. The elimination of the minimal pertinent features is done one by one so that the absolute backward feature depletion is done and ultimately the optimal feature fragment is reaped. A dual combination of SVM and RFE is SVM-RFE. When the training of SVM is done, the feature weights can easily reflect their original contribution to the process of classification and decision-making. As a basis of feature ranking, the weight of a classifier can be utilized. Depending on the weight of the classifier, the deletion of the relatively unimportant features is done slowly unless the features with soaring significance alone are left. For the feature reduction and selection using SVM-RFE technique, the main steps are as follows:

Step 1 Training sample data as input is considered as $D={\left\{d_{1},d_{2},...,d_{n}\right\}}^{T}$ and the label category is assigned as $L={\left\{l_{1},l_{2},...,l_{n}\right\}}^{T}$.
Step 2 The feature set $\alpha =\left\{\lambda_{1},\lambda_{2},...,\lambda_{n}\right\}$ is initialized and the feature set β={} is rearranged.
Step 3 For training the input data, the SVM classifier is utilized and the parameter data of the support vector is expressed as δ=SVMtrain(D,L).
Step 4 The feature cost function is computed as follows:

(10) $$f\left(x\right)=\frac{1}{2}D^{T}M\left(x\right)-\frac{1}{2}D^{T}M\left(-x\right)$$

where a matrix with dent $e_{i}e_{j}K\left(x_{i},x_{j}\right)$, M(−x) is the matrix obtained after rejecting x features. The Kernel function of correlation in between $x_{i}$ and $x_{j}$ is expressed by K.

Step 5 To record the new features, the weight coherent w is utilized as the categorization benchmark of feature significance.
Step 6 Now a new feature order set $\beta =\left\{\beta_{1},\beta_{2},...,\beta_{n}\right\}$ is obtained and the features with the minuscule weight coefficient from the present order collection is removed. Unless the features are deleted, steps 3–5 are repeated.
Step 7 Finally, a group of feature subsets $S_{1}\subset S_{2},...,S_{n}$ is defined, where $S_{i}\left(i=1,2,...,n\right)$ express a subset of the most vital features determined from the feature collection and then use the classifier returns to assess the evaluation index. The classifiers used here KNN, Adaboost, DT, RT, NBC, SVM.

## 3 Proposed technique 2: proposed RR-SRC model

The sparse analysis of measurable signals in a comprehensive manner could be done only by the sparse representation [58]. In the arena of applied pattern recognition, the sparse representation is utilized for classification too. In this sparse rendition, the data matrix $Z=\left[z_{1},z_{2},...,z_{n}\right]$ can be easily split into a linear blending of a few atoms in the dictionary and it is represented as,

(11) Z≈DC

where the dictionary matrix is indicated as D and the coefficient matrix is specified as C. A sufficient conjecture makes DC the sparse representative as a logical assessment of Z. Depending on this postulation, equation (11) can be rewritten as follows:

(12) $$\underset{D,C}{\mathrm{argmin}}{\left\|Z-DC\right\|}_{F}^{2}$$

(13) $$Such\;that,\forall_{i},{\left\|c_{i}\right\|}_{0}\leq l_{0}$$

where the sparsity constraint is represented as $l_{0}$ and $c_{i}$ represent the scattered coefficient vector to indicate $Z_{i}$ over D. K-SVD is utilized to solve equation (12) where the alternate updating of both sparse coding and dictionary are done.

### 3.1 Realm revamping model

**Fig. 3:**
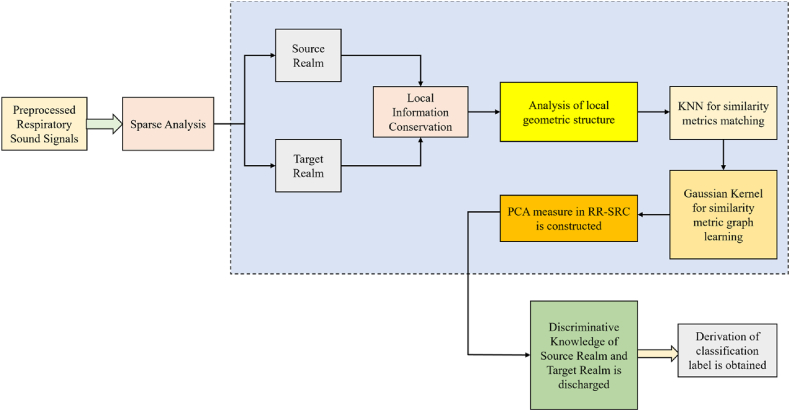
Proposed RR-SRC model.

The respiratory sound data from two different realms Source realm (SR) and Target realm (TR) are considered in this study. There are sufficient labelled samples in the SR $Z^{s}=\left[z_{1}^{s},z_{2}^{s},...,z_{n_{s}}^{s}\right]\in R^{d\times n_{s}}$ and there are limited labelled samples $Z^{t}=\left[z_{1}^{t},z_{2}^{t},...,z_{n_{t}}^{t}\right]\in R^{d\times n_{t}}$, such that the data distribution is expressed as:

(14) $$P\left(Z^{s}\neq Z^{t}\right)\;and\;P\left(Z^{s}|{\tilde{L}}^{s}\right)\approx P\left(Z^{t}|{\tilde{L}}^{t}\right)$$

where the class label collection of samples in source realm is expressed as $Z{\tilde{L}}^{s}$ and the class label collection of samples in target realm is expressed as ${\tilde{L}}^{t}$. The proposed RR-SRC model is elucidated in Fig. 3.

### 3.2 Local information conserved in RR- SRC

The domain discrepancies exist between the SR and TR as the respiratory sound signals are collected from various domains. If the direct adaptation of the existing classifier in SR is done it might accomplish poorly to the novel samples present in TR [59]. To trace the latent and domain-invariant maps along with its subspace $z^{s}$ and $z^{t}$, realm revamping is done by the usage of projection matrices $M^{s}$ and $M^{t}$. The discrepancy present in between the SR and TR can be mitigated with the help of this domain-invariant subspace. A new classifier for TR can be trained in the subspace with the aid of the discriminative comprehensive understanding obtained from labelled samples in SR. To safeguard the class dispensation and the local attributes of training samples, the realm revamping techniques tries very hard to achieve it. By means of considering the local geometric constitution of the samples, the samples in the SR and TR can be indicated specifically in the domain-invariant subspace. By means of using K-nearest neighbor graphs, the similarity matrices $H^{s}$ and $H^{t}$ of SR and TR is constructed. With the help of the following equation, the elements in $H^{s}$ and $H^{t}$ can be determined as follows:

(15) $$H_{i,j}^{s}=\left\{ \begin{array}{c} \exp\left(-\frac{{\left\|Z_{i}^{s}-Z_{j}^{s}\right\|}_{2}}{\sigma }\right)\\ 0 \end{array}\right.\begin{array}{c} if\;Z_{i}^{s}\in KNN\left(Z_{j}^{s}\right)\\ else \end{array}$$

(16) $$H_{i,j}^{t}\left\{ \begin{array}{c} 1\\ 0 \end{array}\right.\begin{array}{c} if\;z_{i}^{t}and\;z_{j}^{t}are\;of\;sameclass\\ else \end{array}$$

where $KNN\left(z_{j}^{s}\right)$ is a collection that accommodates KNN samples of $z_{j}^{s}$.

The resemblance in between the $i^{th}$ and $j^{th}$ samples in SR is expressed by the element $H_{i,j}^{s}$. The Gaussian Kernel is considered in equation (15) as it is one of the most widely utilized similarity metric graph learning. The kernel representation is represented by σ. As there are insufficient number of samples in TR, equation (16) makes sure that the restricted number of samples in TR is allocated with a particular weight. Consequently, the local conserved condition is constructed so that the intra-domain local information is maintained.

(17) $$\min_{M^{s},M^{t}}J_{1}={\sum_{i,j}H_{ij}^{s}\left\|\left(M^{s}Z_{i}^{s}-M^{s}Z_{j}^{s}\right)\right\|}_{2}^{2}+{\sum_{i,j}H_{ij}^{t}\left\|\left(M^{t}Z_{i}^{t}-M^{t}Z_{j}^{t}\right)\right\|}_{2}^{2}$$

Now the Principal Component Analysis (PCA) measure in RR-SRC is constructed. The discriminative knowledge of SR and TR is discharged in subspace projection with the main intention of classification. Based on the PCA measure for SR and TR, the bases of subspaces are considered as $M^{s}$ and $M^{t}$ respectively. Now to preserve the discriminative and comprehensive knowledge in the subspace, the PCA criterion is widely used. The following optimization problem is minimized and represented as:

(18) $$\min_{M^{s},M^{t}}J_{2}=-M^{s}Z^{s}{\left(Z^{s}\right)}^{T}{\left(M^{s}\right)}^{T}-M^{t}Z^{t}{\left(Z^{t}\right)}^{T}{\left(M^{t}\right)}^{T}$$

(19) $$s.t\;M^{s}{\left(M^{s}\right)}^{T}=I\;and\;M^{t}{\left(M^{t}\right)}^{T}=I$$

where the identity matrix is indicated by I. The Laplacian matrices are expressed as $L^{s}=H^{s}-W^{s}$ and $L^{t}=H^{t}-W^{t}$, where the diagonal matrices $H^{s}$ and $H^{t}$ are represented by the constituents in the main diagonal expressed as:

(20) $$W_{i,i}^{s}=\sum_{j}H_{i,j}^{s}\;and\;W_{i,i}^{t}=\sum_{j}H_{i,j}^{t}$$

The term $J_{1}+J_{2}$ is expressed as follows:

(21) $$\min_{M^{s},M^{t}}J_{1}+J_{2}=Tr\left(M^{s}Z^{s}\left(L^{s}-\alpha I\right){\left(Z^{s}\right)}^{T}{\left(M^{s}\right)}^{T}\right)+Tr\left(M^{t}Z^{t}\left(L^{t}-\alpha I\right){\left(Z^{t}\right)}^{T}{\left(M^{t}\right)}^{T}\right)$$

(22) $$s.t\;M^{s}{\left(M^{s}\right)}^{T}=I\;and\;M^{t}{\left(M^{t}\right)}^{T}=I$$

where the parameter of regularization is indicated as α.

### 3.3 RR-SRC model implementation

For the respiratory sound based classification, the two objective functions $J_{1}$ and $J_{2}$ are considered together so that the joint constraint on the PCA and the local information is utilized to enhance the shared dictionary D and the domain-particular projection $\tilde{M}$. Once these are found out, the basic step is utilized to acknowledge the new respiratory sound signal z in TR. Over the dictionary, the sparse coefficient vector α is solved and is expressed as follows:

(23) $$\min_{\alpha }{\left\|\tilde{M}z-D\alpha \right\|}_{2}^{2}+\mu {\left\|\alpha \right\|}_{2}^{2}$$

The sparse coefficient parameter is represented as:

(24) $$\alpha ={\left(D^{T}D+\mu I\right)}^{-1}D^{T}\tilde{M}z$$

The derivation of the classification label of x is obtained as:

(25) $$label\left(z\right)=\underset{i}{\mathrm{argmin}}\left\{{\left\|\tilde{M}z-D\varphi_{i}\left(\alpha \right)\right\|}_{2}^{2}/{\left\|\varphi_{i}\left(\alpha \right)\right\|}_{2}^{2}\right\},i=1,2,...,N$$

where the total number of classes and is indicated as N and the coefficient vectors of the same class of z is denoted by $\varphi_{i}$.

## 4 Proposed technique 3: Hybrid Distance Metric Based VMD with ELM

A novel prediction model dependent on Distance Metric based VMD (DM-VMD) with ELM and other classifiers are proposed here.

Step 1 DM-VMD Decomposition process:

The respiratory sound is initially normalized with the help of equation (26) initially. Then a series of IMFs is decomposed by the normalized with the help of proposed DM-VMD techniques as follows:

(26) $$p_{k}=\frac{p_{k}-p_{\min}}{p_{\max}-p_{\min}}$$

Step 2 In this process, the prediction of IMF is concentrated more. The division of the IMF into two samples such as train sample and test sample are done.
Step 3 For every mode $\left\{IMF_{1}^{\prime },IMF_{2}^{\prime },...,IMF_{k}^{\prime }\right\}$, the ELM or the respective classifier is established and the prediction result is obtained as $Q=\left\{IMF_{1}^{\prime },IMF_{2}^{\prime },...,IMF_{k}^{\prime }\right\}$.
Step 4 The error compensation process happened as the processing of each of the $Q^{*}$ prediction results is done by recursive error correction so that tertiary schemes of recursive error data is formed such as $E_{1},E_{2}$ and $E_{3}$ respectively and it is expressed as:

(27) $$E=E_{1}+E_{2}+E_{3}$$

Step 5 The hybrid reconstruction process is now continued. $Q^{*}=\left\{IMF_{1}^{\prime },IMF_{2}^{\prime },...,IMF_{k}^{\prime }\right\}$ obtained in step (3) and the iterative error data E secured in step (4) are reconstructed so that the prediction can be concluded.
Step 6 The culminating prediction outcome $P_{pre}$ is considered as an output and the hybrid reconstruction equation is considered as follows:

(28) $$P_{pre}=Q^{*}+E$$

**Fig. 4:**
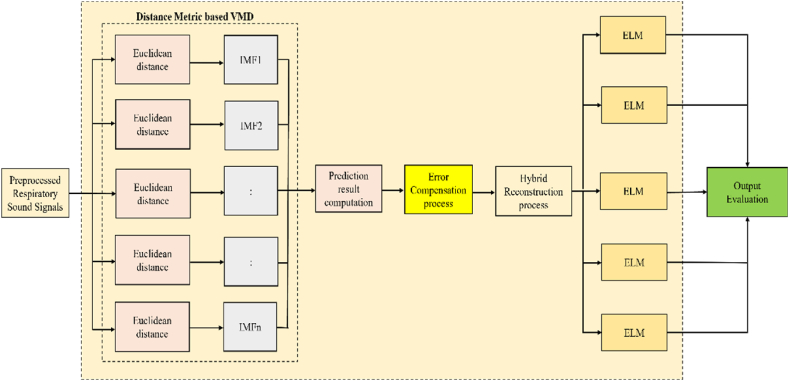
DM based VMD with ELM classifier.

Now the concept of DM VMD-ELM classifier is introduced in detail with the simplified representation elucidated in Fig. 4. Initially, Euclidean distance is utilized and then it is replaced with the other four distance metrics like Manhattan, Chebyshev, Jaccard Index and Sorensen-Dice Index.

### 4.1 VMD module

The respiratory sound data can be decomposed into a procession of IMFs by VMD and every IMF has the internal attributes of respiratory sound or audio [60]. Assuming that the predicted respiratory sound data is D(t). Discovering the quintessential solution of constrained variational prototype is the primary intention of VMD and then d(t) is decomposed into a procession of distinct frequency bands $b_{k}\left(t\right)$ which is nothing but the necessary IMF constituents. The original signal is equal to the sum of each mode sequence and for every mode sequence, the sum of its estimated bandwidth is generally small. Such as model can be represented as:

(29) $$\left\{\begin{array}{l} \min_{\left\{b_{k}\right\},\left\{c_{k}\right\}}\left\{\sum_{k=1}^{K}{\left\|\partial_{t}\left[\left(\delta \left(t\right)+\frac{j}{\pi t}\right)\times b_{k}\left(t\right)\right]e^{-jc_{k}t}\right\|}_{2}^{2}\right\}\\ s.t.\sum_{k=1}^{K}b_{k}\left(t\right)=d\left(t\right) \end{array}\right\}$$

here the mode sequence is represented by $b_{k}\left(t\right)$ and the corresponding center frequency is denoted by $c_{k}$. The transformation of the above equation into an unconstrained one is done by means of introducing Lagrange multiplier λ and balance parameters of the data-fidelity constraint α.

(30) $$L\left(\left\{b_{k}\right\},\left\{c_{k}\right\},\lambda \right)=\alpha \sum_{k=1}^{K}{\left\|\partial_{t}\left[\left(\delta \left(t\right)+\frac{j}{\pi t}\right)\times b_{k}^{\left(t\right)}\right]e^{-jc_{k}t}\right\|}_{2}^{2}+{\left\|d\left(t\right)-\sum_{k=1}^{K}b_{k}\left(t\right)\right\|}_{2}^{2}+\left\langle \lambda \left(t\right),d\left(t\right)-\sum_{k=1}^{K}b_{k}\left(t\right)\right\rangle$$

By means of alternatively directing methods of multipliers, equation (30) is solved. In the spectral domain, all the required modes obtained from solutions are expressed as:

(31) $${\hat{b}}_{k}\left(c\right)=\frac{\hat{d}\left(w\right)-\sum_{k=1}^{K}{\hat{b}}_{k}\left(c\right)+\frac{\hat{\lambda }\left(c\right)}{2}}{1+2\alpha {\left(c-c_{k}\right)}^{2}}$$

where at the center of gravity, $c_{k}$ is computed to express the respective power spectrum of the modes.

Thus, to the VMD algorithm, the embedding of wiener filter is done and it is more vigorous to noise and sampling.

(32) $$c_{k}=\frac{\int_{0}^{\infty }c{\left|{\hat{b}}_{k}\left(c\right)\right|}^{2}dc}{\int_{0}^{\infty }{\left|{\hat{b}}_{k}\left(c\right)\right|}^{2}dc}$$

### 4.2 Distance metrics based VMD

The mode number K must be present by VMD. If the K value is improper, then it will accompany to an enormous bias in the reconstructed signal. Selection of the exact K values play an input role on the correct decomposition of the respiratory sound signal. To deal with this problem, DM-VMD is proposed which uses distance metrics like Euclidean distance, Manhattan distance, Chebyshev distance, Jaccard Index and Sorensen-Dice Index to determine the K values [61]. To achieve better prediction results, IMF is established with ELM. To assess the similarity between the two variables, the method utilized is DM. The similarity between the variables would be greater if the values computed to close to 1. If variables $x=\left(x_{1},x_{2},...,x_{n}\right)$ and $y=\left(y_{1},y_{2},...,y_{n}\right)$ are assumed, then the distance metric computed x and y would be follows accordingly.

1) Euclidean distance [61]: This distance measure can be expressed well as the length of a segment which can connect 2 points easily. The mathematical expression is straightforward as the calculation of the distance is done from the cartesian coordinates of the points utilizing the Pythagorean theorem.

(33) $$D\left(x,y\right)=\sqrt{\sum_{i=1}^{n}{\left(x_{i}-y_{i}\right)}^{2}}$$

2) Manhattan distance [61]: Often termed as city block distance, it computes the distance between the real-valued vectors. Here there is no involvement of the diagonal movement in computing the distance. The distance between two vectors if the movement is involved with only right angles is considered here.

(34) $$D\left(x,y\right)=\sum_{i=1}^{K}\left|x_{i}-y_{i}\right|$$

3) Chebyshev distance [61]: Along any co-ordinate dimension, the greatest difference between 2 vectors is defined by the Chebyshev distance. The maximum distance along one axis is assessed by Chebyshev distance and is measured by:

(35) $$D\left(x,y\right)=\underset{Chebyshev}{\max_{i}}\left(\left|x_{i}-y_{i}\right|\right)$$

4) Jaccard Index [61]: The analogy and distinctiveness of sample sets can be calculated by Jaccard Index. The convergence or the intersection size divided by the size of the number of sample sets gives us the JI. A simple subtraction of the JI from 1 is used to compute the Jaccard distance and is indicated as:

(36) $$D\left(x,y\right)=1-\frac{\left|x\cap y\right|}{\left|y\cup x\right|}$$

5) Sorensen-Dice Index [61]: It is quite similar to JI and it is often seen as the percentage of overlap between 2 sets having a value between 0 and 1 and is indicated as:

(37) $$D\left(x,y\right)=\frac{2\left|x\cap y\right|}{\left|x\right|+\left|y\right|}$$

By means of decomposing the authentic signal y, to get the reconstructed signal $\hat{y}$, the IMFs are utilized. The final mode is determined by DM-VMD by means of computing the DM values of y and $\hat{y}$. The DM values will surge if there is an increase in K value. During the experiments it is understood that the DM value reaches in the range of 0.85–0.99 as only when it reaches a peak value, it can attain stability, thereby indicating that the decomposition of the signal has been completed by this time. The threshold is set to a maximum of 0.99 is our experiment. The explanation for DM-VMD is given in Algorithm 1 as follows.

**Algorithm 1:**
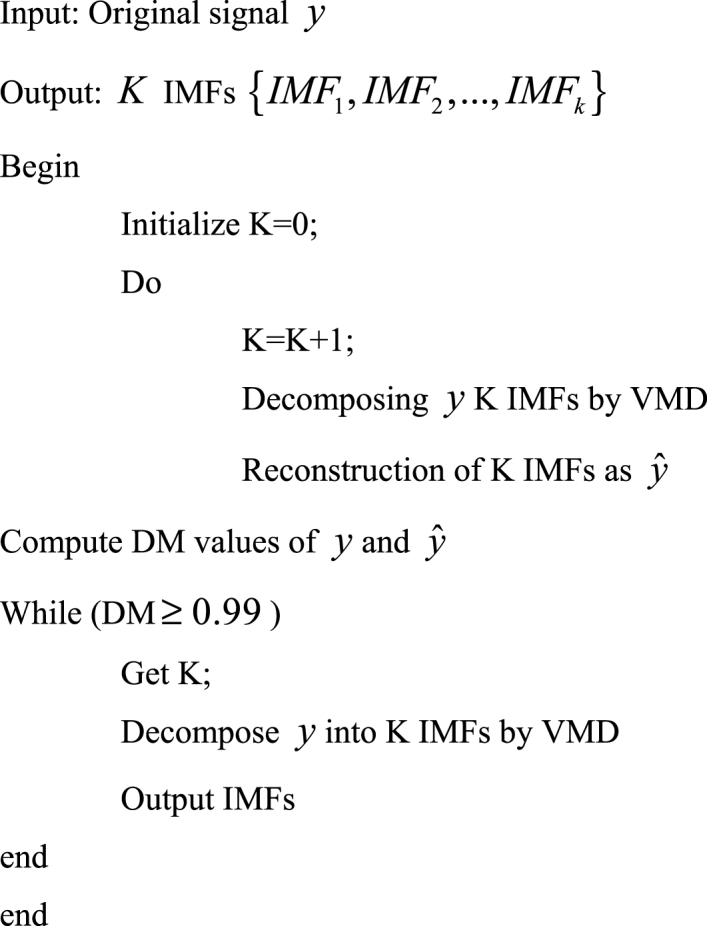
Explanation for DM-VMD.

### 4.3 ELM model

**Fig. 5:**
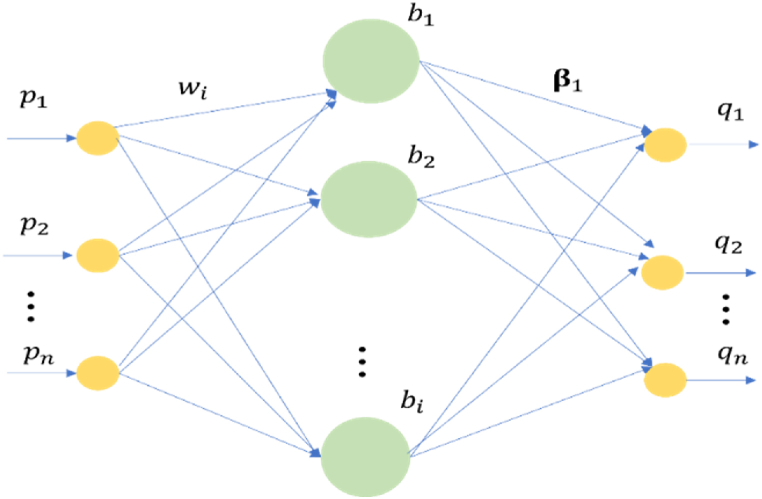
Structure of an ELM network.

The IMFs which are decomposed by DM-VMD are directly catered into the ELM so that the temporal features are learned thoroughly. ELM is a single hidden layer feed forward neural network technique which has a magnificent executing action [62]. The simple structure of an ELM network is expressed in Fig. 5.

In Fig. 5, $p_{1},...,p_{n}$ is indicated as the input vector and $q_{1},..,q_{m}$ is denoted as output vector. The weights interconnecting the input layer and hidden layer are represented by $w_{i}={\left[w_{i1},w_{i2},...,w_{in}\right]}^{T}$. The weights linking the hidden layer and output layer is indicated by $\beta_{i}={\left[\beta_{i1},\beta_{i2},...,\beta_{in}\right]}^{T}$. If an 'n' arbitrary sample is assumed, the $i^{th}$ one is specified as $\left(p_{i},t_{i}\right),p_{i}={\left[p_{i1},p_{i2},...,p_{in}\right]}^{T}\in R^{n},t_{i}={\left[t_{i1},t_{i2},...,t_{in}\right]}^{T}\in R^{m}$, then a single hidden layer neural network with 'L' hidden layer node is indicated as:

(38) $$\sum_{i=1}^{L}\beta_{i}g\left(w_{i}.p_{j}+b_{i}\right)=q_{j},j=1,2,...,n$$

where the excitation function is indicated as g(x), and the bias of the $i^{th}$ hidden layer neuron is expressed as $b_{i}$.

The output error is minimized and it is the main learning objective of single hidden layer neural network, i.e, with the presence of $w_{i},b_{i},\beta_{i}$, the equation can be specified as:

(39) $$\sum_{i=1}^{L}\beta_{i}g\left(w_{i}.p_{j}+b_{i}\right)=t_{j},j=1,2,...,N\text{,}$$

For simplicity reasons, the above equation can also be written and defined as follows:

(40) Hβ=T

where the output matrix of hidden layer neural network is expressed as H, the output weight matrix is expressed as β and the aspired output matrix is expressed as T.

(41) $$H=\left[\begin{array}{ccc} g\left(w_{1}p_{1}+b_{1}\right) & ...... & g\left(w_{L}p_{1}+b_{L}\right)\\ : & ...... & :\\ g\left(w_{1}p_{N}+b_{1}\right) & ...... & g\left(w_{L}p_{N}+b_{L}\right) \end{array}\right]$$

(42) $$\beta =\left[\begin{array}{c} \beta_{1}\\ :\\ \beta_{L} \end{array}\right]$$

(43) $$T=\left[\begin{array}{c} T_{1}\\ :\\ T_{L} \end{array}\right]$$

The main intention of training the single hidden layer neural network can be achieved if ${\hat{w}}_{i},{\hat{b}}_{i},{\hat{\beta }}_{i}$ is found out, thereby the following is achieved as:

(44) $$\left\|H\left({\hat{w}}_{i},{\hat{b}}_{i}\right){\hat{\beta }}_{1}-T^{\prime }\right\|=\min_{w,b,\beta }\left\|H\left(w_{i},b_{i}\right)\beta_{i}-T^{\prime }\right\|,i=1,2,...,m$$

The above equation is similar to solve the minimum loss function as follows:

(45) $$E={\sum_{j=1}^{N}\left[\sum_{i=1}^{L}\beta_{i}g\left(w_{i}.p_{i}+b_{i}\right)-t_{i}\right]}^{2}$$

For the hidden layer the output matrix H won't be changed when $w_{i}$ and $b_{i}$ are assessed. The least square explication $\beta^{T}$ of Hβ=T is similar or equal to the training process of ELM currently and is expressed as:

(46) $$\hat{\beta }=H^{+}T$$

where for the hidden layer node output matrix, the Moore-Penroe generalized inverse is expressed as $H^{+}$.

## 5 Proposed technique 4: HHO-SFPDE based classification

### 5.1 Harris Hawks Optimization

A bionic optimization algorithm motivated by the prey-predator behaviour of the Harris Hawks is HHO algorithm [63]. It has been utilized to deal with complex optimization problems also. The overall energy of the scenario along with the escape energy level of the prey is heavily influencing this algorithm. At various stages, in order to imitate and replicate Harris Hawk's exploration and exploitation features, distinct position update strategies are used. It is a non-gradient and swarm algorithm comprising of three stages such as exploration, the transit stage from exploration to exploitation and finally the exploitation stage. The initial parameters are easy to determine and control as the HHO structure is quite flexible. In terms of computational efficiency, the accomplishment of HHO is quite superior to other algorithms and so it is used here. Due to its relatively easy usage and implementation along with the smaller number of initial controlling parameters HHO is widely used.

#### 5.1.1 Exploration phase

A certain place is randomly inhabited by Harris Hawks and then one of the two master plans is chosen to find the prey ‘Rodent’ based on the random state and is represented as

(47) $$X\left(t+1\right)=\left\{ \begin{array}{c} X_{rand}\left(t\right)-r_{1}\left|X_{rand}\left(t\right)-2r_{2}X\left(t\right)\right|,n\geq 0.5\\ \left[X_{rodent}\left(t\right)-X_{p}\left(t\right)\right]-r_{3}\left[l_{b}+r_{4}\left(u_{b}-l_{b}\right)\right],n<0.5 \end{array}\right.$$

where X(t) indicates the individual position at the present iteration and X(t+1) stipulates the individual position at the next iterative loop. The randomly chosen individual position is constituted as $X_{rand}$ and the number of iterative loops is indicated as t. The prey position is mentioned by $X_{rodent}\left(t\right)$. The random numbers present in the span of [0,1] is represented by $r_{1},r_{2},r_{3},r_{4}$ and q respectively. $l_{b}$ indicates the lower bound of search space and $u_{b}$ specifies the upper bound of search space. The individual's average position is represented by $X_{p}\left(t\right)$ and is represented as:

(48) $$X_{p}\left(t\right)=\sum_{k=1}^{P}X_{k}\left(t\right)/P$$

where the position of orientation for the $k^{th}$ individual in the population is expressed as $X_{k}\left(t\right)$. The population size is expressed as

(49) $$A_{p}=X_{rodent}\left(t\right)-E\left|D.X_{rodent}\left(t\right)-X\right|\text{.}$$

Transit stage from exploration stage to exploitation stage:

In the escape phase, depending on the outright merit of the prey's escape energy, the determination of the transit from exploration to exploitation is done. It enters the exploitation stage if the value of the absolute escape energy |E| is substantially appreciable and higher than 1 and it enters the exploration stage if the value of the absolute escape energy |E| is less than 1. The definition of escape energy is expressed as follows:

(50) $$E=2E_{0}\left(1-\frac{t}{T}\right)$$

where T represents the maximum iteration number, t indicates the current number of iterations and $E_{0}$ represents a random number in between [-1,1].

#### 5.1.2 Exploitation phase

Based on the random number r and the escape energy |E| the exploitation episode is determined as a piecewise function. There are four important conditions here:

Firstly, if r≥0.5 and the value of |E| is as follows: 0.5≤|E|<1, then the updation procedure of Harris Hawks is done as follows:

(51) $$X\left(t+1\right)=\Delta X\left(t\right)-E\left|D.X_{rodent}\left(t\right)-X\left(t\right)\right|$$

where the range number in the range of [0,2] is indicated by D. The basic difference or contrast between the position of the prey and the position of the present individuals is expressed as follows:

(52) $$\Delta X\left(t\right)=X_{rodent}\left(t\right)-X\left(t\right)$$

Secondly, if r>0.5 and the value of |E| is less than 0.5, then the updation of the Harris Hawks position is done as follows:

(53) $$\Delta X\left(t+1\right)=X_{rodent}\left(t\right)-E\left|\Delta X\left(t\right)\right|$$

Thirdly, if r<0.5 and the value of |E| is as follows: 0.5≤|E|<1, then the updation of Harris Hawk is observed as:

(54) $$X\left(t+1\right)=\left\{ \begin{array}{c} A.f\left(A\right)<f\left(X\left(t\right)\right)\\ B.f\left(B\right)<f\left(X\left(t\right)\right) \end{array}\right.$$

where f() is represented as the fitness function, A and B are indicated as follows:

(55) $$\left\{ \begin{array}{c} A=X_{rodent}\left(t\right)-E\left|D.X_{rodent}\left(t\right)-X\left(t\right)\right|\\ B=A+V\times LF\left(DIME\right){}^{\circ} \end{array}\right.$$

where DIME represents the problem dimension. The vector of DIME whose elements are just random numbers in the span of [0.1] is represented as V. LF(DIME) represents a DIME dimensional Levy's flight vector expressed as follows:

(56) $$\left\{ \begin{array}{c} LF\left(\right)=\frac{0.01\lambda g\times \sigma }{{\left|h\right|}^{\frac{1}{\beta }}}\\ \sigma =\{{\frac{T\left(1+\beta \right)\sin\left(\frac{\pi \beta }{2}\right)}{\beta T\left(\frac{1+\beta }{2}\right)2^{\frac{\beta -1}{2}}}\}}^{\frac{1}{\beta }} \end{array}\right.$$

where the 2 random number of DIME are reported by g and h, and it is usually in the range of [0,1] and 0<β<2 is a standard index and its value is assumed to be from 0.5 to 1.5 respectively.

Fourthly, when r<0.5 and |E| <0.5, the updation of position of Harris Hawks is as follows:

(57) $$X\left(t+1\right)=\left\{ \begin{array}{c} A_{P},f\left(A_{P}\right)<f\left(X\left(t\right)\right)\\ B,f\left(B\right)<f\left(X\left(t\right)\right) \end{array}\right.$$

where

(58) $$A_{p}=X_{rodent}\left(t\right)-E\left|D.X_{rodent}\left(t\right)-X_{p}\left(t\right)\right|$$

**Fig. 6:**
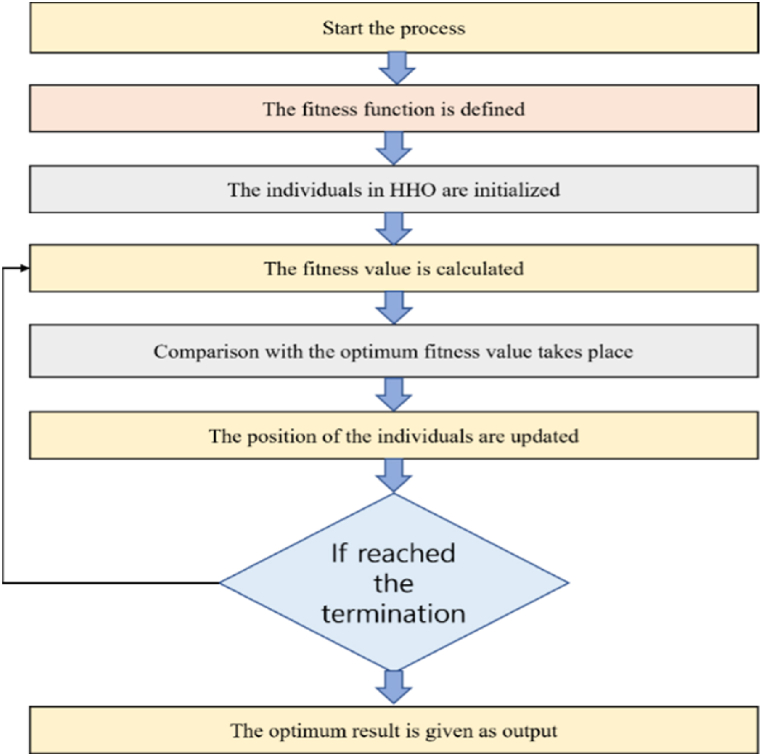
HHO based algorithm flowchart.

The updation of various positions are done as per the above algorithm based on the four random states. Ultimately, the fitness merit of all individuals is computed. The better individuals replan the prey if the fitness value is one step ahead and progressing than that of the prey and once this is done, the next iteration begins. Unless the termination condition is satisfied, this procedure continues. The illustration of the HHO based algorithm is expressed in Fig. 6.

### 5.2 SFPDE algorithm

**Fig. 7:**
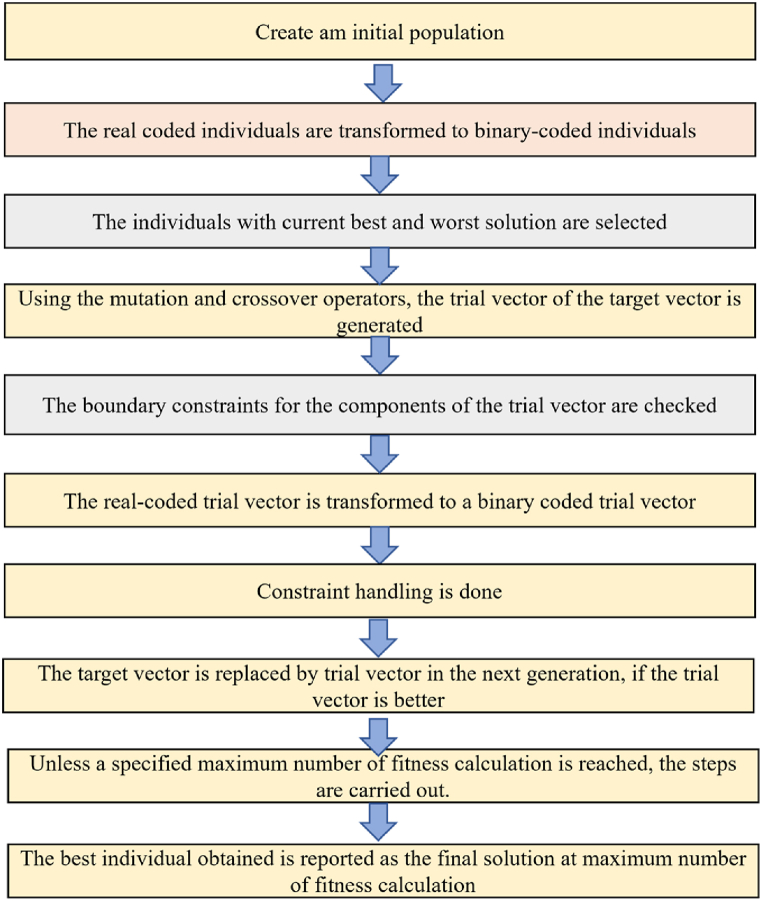
Scaling Factor based Pliable Differential Evolution algorithm.

One of the fastest and simplest techniques performing well on different problems is DE [64]. Like GA, it is a population dependent stochastic search approach with 3 operators such as crossover, mutation, and selection. DE heavily relies on mutation operation while GA heavily relies on crossover so that better solutions can be constructed. The primary functioning is dependent on the contrast dissimilarities of randomly sampled matches of solutions present in the entire population. The mutation operation is used by the algorithm as a hunt tactic technique. In order to mediate the hunt towards better neighborhoods in the search space, the selection operation is used. Generation of a novel trial vector is the principal idea of the DE scheme. To generate a mutant vector, various differential vectors procured from the dissimilarities of diversely selected random parameter vectors are appended to the target vector when the mutation is incorporated. To the target vector, the obtained mutant vector is crossover recombined and so a trial vector is produced. The target vector is restored with the trial vector if a superior fitness value is obtained by the trial vector rather than the target vector. In this particular implementation, a scaling factor based pliable DE algorithm is utilized and the flowchart representation of it is shown in Fig. 7.

#### 5.2.1 Parameter vector initialization

For an n-dimensional original parameter space, a global optimum point is searched by DE. An arbitrarily initialized population of NP n-dimensional real-valued parameter vectors are considered initially. A candidate explication is formed by each vector to the multi-dimensional optimization issue. In DE, the successive generations are indicated by $t=0,1,...,t_{\max}$. Initially, the $q^{th}$ vector of a particular population at the present generation t is specified as:

(59) $$X_{q}\left(t\right)=\left[x_{q,1}\left(t\right),x_{q,2}\left(t\right),...,x_{q,n}\left(t\right)\right]$$

where $x_{q,i}\left(t\right)$ denotes the $i^{th}$ component of the $q^{th}$ vector in the population, i=1,2,...,n,q=1,2,...,NP.

The entire search space should be chosen by the commencing population at (t=0) to the best possible extent by means of randomizing the initial individuals uniformly prescribed by the minimum parameter bound $X^{\min}=\left[x_{1}^{\min},...,x_{n}^{\min}\right]$ and the maximum parameter bound $X^{\max}=\left[x_{1}^{\max},...,x_{n}^{\max}\right]$. The prime value of the $i^{th}$ constituent of the $q^{th}$ individual $X_{q}\left(t\right)$ at a particular generation t=0 is represented by:

(60) $$x_{q,i}\left(0\right)=x_{i}^{\min}+\left(x_{i}^{\max}-x_{i}^{\min}\right).\mathrm{ran}d_{q,i}$$

where a random number with uniform distribution is represented by $\mathrm{ran}d_{q,i}$ and it lies in the range of 0 and 1. For every constituent i∈{1,2,...,n} of the $q^{th}$ vector, it is initiated independently.

#### 5.2.2 Implementation of a self-pliable scaling factor for mutation

A mutant vector is produced with respect to every individual by the mutant operation after the initialization process [65]. At generation t, for each and every target vector $X_{q}\left(t\right)$, its respective mutant vector $Y_{q}\left(t\right)$ can be obtained as follows:

(61) $$Y_{q}\left(t\right)=X_{q1}\left(t\right)+S\cdot \left(X_{q2}\left(t\right)-X_{q3}\left(t\right)\right)$$

where the randomly chosen individuals are represented as $X_{q1}\left(t\right),X_{q2}\left(t\right)\text{,}$ and $X_{q3}\left(t\right)$ respectively from the identical generation with $q\neq q_{1}\neq q_{2}\neq q_{3}$. The scaling factor is indicated as S and it is a positive constant. The amplification of the difference vector is influenced by this scaling factor S and is expressed as:

(62) $$X_{q2}\left(t\right)-X_{q3}\left(t\right)$$

The prevalent setting for this factor is S∈[0,2]. Some researchers have used the S∈[0.5,1] too. One of the essential factors influencing the DE's accomplishment is the scaling factor S. The choosing of this value S is quite difficult as it is usually problem dependent. A variable scaling factor was proposed by some researchers in the past to improve the execution of DE. The exploration (global) and exploitation (local) ability of the population is balanced by the scaling factor S. For the purpose of exploration, there is a huge contribution of an enormous mutation factor but the drawback is that it takes an extended period of time for convergence purposes. To achieve a fast convergence of the population, a small scaling factor is enough but it progresses to local optimal sometimes. Therefore, the need for a self-adaptive DE algorithm comes into picture. For all the individuals, if the same scaling factor is introduced by means of ignoring the differences of the performance of individuals, then it is not a good model. In a dynamic manner, all the individual changes its position during the search and so a different situation is forced by the respective individual in a specific environment. Therefore in between the global and local search abilities, various tradeoffs are achieved. For every individual, there is a dynamic adaptation of the scaling factor and it is done by means of instigating a measure termed affinity index. At a 'tth' iteration, the closeness of a distinctive answer of individuals is characterized to the global solutions of population. Depending on this, the adjustment of the scaling factor values could be done easily. The mutation strategy for this purpose is expressed by:

(63) $$Y_{q}\left(t\right)=X_{q}\left(t\right)+S_{q}\left(t\right).\left(X_{q1}\left(t\right)-X_{q2}\left(t\right)\right)$$

In the $t^{th}$ iteration, to compute the scaling factor for the $q^{th}$ target vector, the closeness parameter (CP) is expressed as follows:

(64) $$CP_{q}\left(t\right)=\frac{fit\left(X_{q}\left(t\right)\right)-fit\left(X_{worse}\left(t\right)\right)}{fit\left(X_{best}\left(t\right)\right)-fit\left(X_{worst}\left(t\right)\right)}$$

where $X_{best}\left(t\right)$ indicates the finest solution of the population at the iterative loop t and the worst solution of the population is indicated by $X_{worst}\left(t\right)$ at the iteration t.

(65) $$fit\left(X_{best}\left(t\right)\right)=\max_{q\in \left\{1,...,NP\right\}}\left\{fit\left(X_{q}\left(t\right)\right)\right\}\;and\;fit\left(X_{worst}\left(t\right)\right)=\min_{q\in \left\{1,...,NP\right\}}\left\{fit\left(X_{q}\left(t\right)\right)\right\}$$

where the fitness function is represented by fit(⋅).

It can be finalized that if the value of $CP_{q}\left(t\right)$ is small, then it implies that the $q^{th}$ individual is far away from the global best solution and therefore there is an urgent need for a strong global exploration. If the value of $CP_{q}\left(t\right)$ is big, then it implies that the $q^{th}$ individual is very close to the global best solution and so a secure and powerful local exploitation is needed. For every target individual, at $t^{th}$ iteration the dynamic adaption of the scaling factor value is represented as follows:

(66) $$S_{q}\left(t\right)=\frac{1}{1+\tanh\left(2\cdot CP_{q}\left(t\right)\right)}$$

where the hyperbolic tangent function is represented as tanh(z)..

(67) $$\tanh\left(z\right)=\frac{\exp\left(z\right)-\exp\left(-z\right)}{\exp\left(z\right)+\exp\left(-z\right)}$$

In our work, it is assumed that $0.5<S_{q}\left(t\right)\leq 1$.

#### 5.2.3 Crossover

Once the mutant vector is generated after mutation, a cross over operation makes its presence and comes into effect so that the prospective assortment of the population is enhanced. To configure the trial vector $Z_{q}\left(t\right)$, the mixing of its constituents with the target vector $X_{q}\left(t\right)$ is done by the mutant vector. Two types of crossover techniques are used by DE algorithm such as exponential and binomial. In this paper, binomial crossover is widely used and so a binomial distribution is obtained by the number of specific parameters attained from the mutant and is expressed as:

(68) $$Z_{q,i}\left(t\right)=\left\{ \begin{array}{c} y_{q,i}\left(t\right),if{rand}_{q,i}\leq CR\;or\;i=i_{rand}\\ y_{q,i\left(t\right),otherwise} \end{array}\right.$$

where the $i^{th}$-dimensional components of the vectors are expressed as $y_{q,i}\left(t\right)$ and $z_{q,i}\left(t\right)$ respectively. The predefined crossover probability is denoted as CR and is in the specific range of (0,1) which can be fixed/dynamic. The random selection of the number from the index sector is represented as $i_{rand}$ and it makes sure that the trial vector $Z_{q}\left(t\right)$ is quite discrete and distinct from the actual solution $X_{q}\left(t\right)$. To generate a trial vector, the CR play a vital role as it helps to control the recombination of both vectors (mutant and target).

#### 5.2.4 Selection

To assess whether the target could manage and survive to the succeeding generation at the condition t=t+1, selection process is used. For all the trial vectors, the evaluation of the objective of the objective function values is done. Then the performance of a selective operation is done. The choice between the target and corresponding trial vector is made by the selection operator. Then the vector with a high or better fitness value becomes a member of the next generation and is expressed as:

(69) $$X_{q}\left(t+1\right)=\left\{ \begin{array}{c} Z_{q}\left(t\right),\begin{array}{cc} if & f\left(Z_{q}\left(t\right)\right)\geq f\left(X_{q}\left(t\right)\right) \end{array}\\ X_{q}\left(t\right),\begin{array}{cccc} & & & \begin{array}{cc} & otherwise \end{array} \end{array} \end{array}\right.$$

The corresponding target is restored by the trial vector yielding a better value in the succeeding generation, or else it is preserved in the population. Therefore, the population never deteriorates as it either gets better or remains the same with respect to fitness.

#### 5.2.5 Binarization, constraint handling and stopping criterion

An amended version of DE is binary DE which usually works in the binary search spaces. The conversion of the real value into the binary space is done with the help of binary DE engaging the following rule:

(70) $$x_{q,i}\left(t+1\right)=\left\{ \begin{array}{c} 1,if\begin{array}{cc} \mathrm{ran}d_{q,i}<sigm\left(x_{q,i}(t+1\right) & \end{array}\\ 0,\begin{array}{cccc} & & & \begin{array}{ccc} & & \begin{array}{cc} & otherwise \end{array} \end{array} \end{array} \end{array}\right.$$

When a consistently dispensed random number lies in between 0 and 1 it is represented as $\mathrm{ran}d_{q,i}$. sigm(z) is denoted as the sigmoid function and is specified as follows:

(71) $$sigm\left(z\right)=\frac{1}{1+\exp\left(-z\right)}$$

Mapping the interval $\left[x_{i}^{\min},x_{i}^{\max}\right]$ for every i∈{1,2,...,n} can be done effortlessly with the aid of sigmoid function.

As far as the constraint handling is concerned, the newly generated solution after the initialization, mutation, cross over and binarization may not satisfy the constraint. A famous function to progress and estimate a constrained issue into an unconstrained one is done with the help of penalty method in our work. By means of punishing the infeasible solutions and favoring the feasible solutions, the strategy is quite easy to handle the constraints. Finally, unless the stopping criterion is satisfied, the mutation, crossover and selection process will continue. Once the maximum iteration number is outstretched, then the termination of the DE algorithm takes place and hence the best solution is considered as the output. If the best solution is not given as output, then the updation process (mutation, crossover, and selection) continues.

#### 5.2.6 Parameter settings

The population size (NP), scaling parameter (S) along with the crossover rate (CR) are the most important parameters that influences the performance of DE. In the proposed algorithm, throughout the evolution process, the population size is determined as 100 and it is maintained for the entire experiment. In order to get the global solution with a soaring probability, the merit of NP can be increased. If the merit of NP is high, then the number of fitness assessment too is also high. In the current population, the number of mutating elements is determined by the crossover rate as it is nothing but the likelihood of blending between the trial and target vectors. In order to speed up the convergence, a large CR is used. In our experiment, we have tried the CR in the range of 0.2–0.9 and the best value is selected as 0.7 as it gave good results. Other important formulae are as $t_{\max}=1000$, $x_{i}^{\min}=-2$ and $x_{i}^{\max}=2$ for all i∈{1,2,...,n}.

**Fig. 8:**
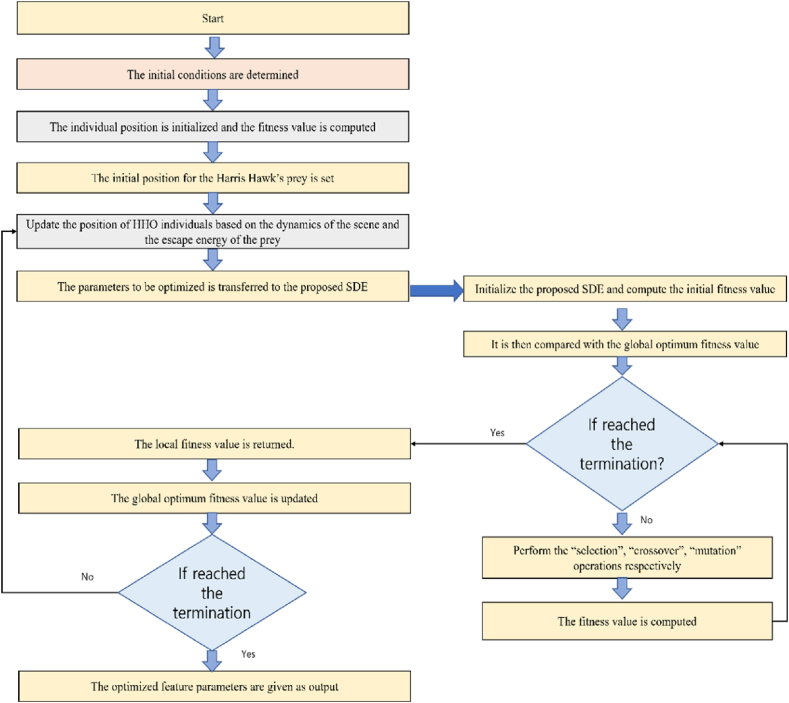
Proposed HHO-SFPDE algorithm flowchart.

The illustration of the proposed HHO-SFPDE algorithm is expressed in Fig. 8. The controlling parameters of the proposed SFPDE algorithm are optimized by the HHO algorithm. The main implementation procedure is as follows: The determination of the initial conditions is done such as sample data, maximum iteration of HHO, population size, number of optimized parameters with upper bound, total number of optimized parameters with lower bound, iteration of SFPDE algorithm, scaling factor along with the termination set of HHO-SFPDE algorithm. The fitness value is then computed once the initialization of individual points is done. The individual positions are randomly initialized with the aid of upper and lower bounds and then utilizing the chosen fitness function, the computation of the fitness value is done. Different outcome basis such as Mean Square Error (MSE), Root Mean Square Error (RMSE) and Mean Absolute Error (MAE) can be utilized as fitness functions and in our experiment, the MSE is implemented. The parameters which are supposed to be enhanced are nothing but the decision variables. The HHO algorithm is maneuvered to randomly create their inception values and then it is merged with the SFPDE algorithm. The initial position of the HHO is set. The position of HHO individuals is then updated and is dependent on both the scene dynamic and the escape energy level of the prey. The strategy of position updation of Harris Hawk's individual is done and thus a sequential generation of many HHO individuals are obtained. A collection of potential optimized parameters is indicated by every Harris Hawk's individual. The transferring of the individual values of the HHO is done to the inner loop of the proposed SFPDE algorithm and it acts as controlling parameters. Once it is transferred to the SFPDE algorithm, the generation of individuals are done and the computation of the fitness values are done accordingly. The operations of the SFPDE take place until the stopping criteria is pleased. The fitness values are then obtained sequentially once the termination of the inner loop is done and finally the best fitness value of the current generation is obtained.

The termination or the stopping standard for the inner loop and outer loop are expressed as follows. For the inner loop, a highest number of iterations should be attained by the SFPDE algorithm. The inner loop termination can happen if the single generation error is less than $10^{-8}$. Until the termination condition of the outer loop is triumphant, the procedural steps are repeated. If the satisfaction of the termination conditions of the outer loop are done, then it implies that the optimal feature parameters are obtained by the SFPDE algorithm. For the outer loop, the termination criteria are that the highest number of iterations should be reached by the HHO algorithm. The fitness value differences between various generations should be less than $10^{-8}$ once the iteration process is over. Once the stopping condition of the outer loop is satiated then it implies that the optimal feature parameters are obtained through the HHO-SFPDE algorithm.

## 6 Proposed technique 5: dimensionality reduction techniques with metaheuristic based Support Vector Classification and Sparse Autoencoders

**Fig. 9:**
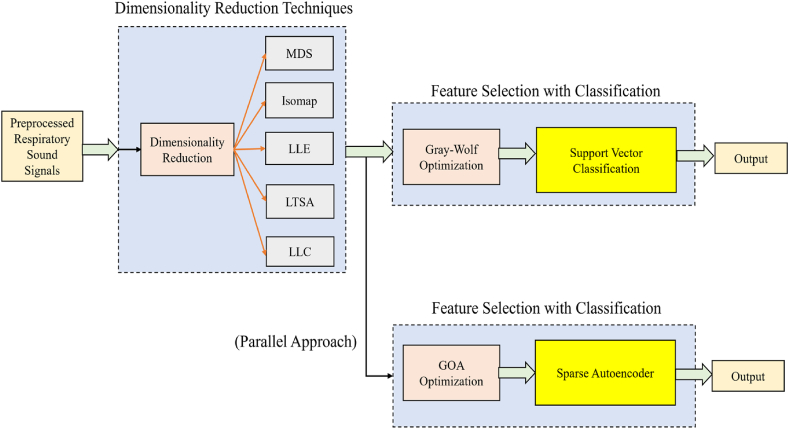
Dimensionality Reduction with Metaheuristic based Classification.

The non-linear dimension reduction done in this work is explained in detail as follows. The respiratory sound dataset is specified in a n×D matrix P which comprises of m data vectors $p_{i}\left(i\in \left\{1,2,...,n\right\}\right)$ with a dimensionality D. An inherent dimensionality 'd' is possessed by this dataset (where d<D or even d≪D). The inherent dimensionality implies that the positions in the dataset P are placed on a manifold which has a dimensionality d, that is eventually implanted in the D-dimensional space. The dataset P with dimensionality D is transformed into a brand new dataset Q with dimensionality d by the dimensionality mitigation schemes, while the data geometry of is retained as much as possible. For the dataset P, both the intrinsic dimensionality d and the data geometry of the data manifold are generally not known. By assuming and analyzing some properties of the data, the dimensionality reduction problem can be solved. In this work, the high-dimensional datapoint is represented by $p_{i}$, where $p_{i}$ indicates the $i^{th}$ row of the D-dimensional data matrix P. The representation of $q_{i}$ is used to indicate the low-dimensional counterpart of $p_{i}$, where $q_{i}$ is the $i^{th}$ row of the d- dimensional data matrix Q. The dimensionality reduction techniques used here are Multidimensional Scaling (MDS) [66], Isometric Mapping (Isomap) [67], Local Linear Embedding (LLE) [68], Local Tangent Space Alignment (LTSA) [69], and Local Linear Coordination (LLC) [70]. Fig. 9 shows the illustration of the dimensionality reduction techniques with GWO-SVC in addition to the GOA with Sparse Autoencoders.

### 6.1 Gray Wolf Optimization based SV classification

One of the famous heuristic group optimization techniques is GWO [71]. It is an optimized search technique where the social hierarchy of gray wolf is simulated emphasizing on the different ways of preying on its prey. It is quite easy to realize, has a few parameters and has a strong convergence performance. SVM is usually used to solve multiclassification problems and in this work, it has been combined with it. A very large interval is defined in the feature space of this classifier and the learning master plan of SVM is to enhance the interval. For nonlinear regression and soft computing problems, SVM algorithm is widely used and here in our experiment to solve the respiratory sound classification, the algorithm used by SVM is SVC. The penalty coefficient c along with the kernel function parameter g influences the classification performances of the SVC model. The SVC parameters are upgraded with the aid of GWO algorithm so that the best classification parameter c and g are found out, so that the GWO-SVC model could be a versatile one. The input steps for the parameter optimization are as follows:

1) The three various categories of primitive wolves are α,β and γ with the similar scale created from achievable regions $W=\left\{w_{1},w_{2},...,w_{n}\right\}$.
2) The orientation of the native wolves is initialized.
3) In the entire population, the fitness value μ of the gray wolf individuals is found out.
4) The optimal fitness is expressed as c and suboptimal fitness are defined as g respectively.
5) For the top three, the fitness is selected and then the corresponding gray wolf is set to α,β and γ respectively.
6) The gray wolf position is used constantly when it preys on the prey.
7) The sub-ordinate wolves are then updated and the updation function is as follows:

(72) $$\left\{ \begin{array}{c} U_{\alpha }=\left|W\left(t\right)-H_{1}W_{\alpha }\right|\\ U_{\beta }=\left|W\left(t\right)-H_{2}W_{\beta }\right|\\ U_{\gamma }=\left|W\left(t\right)-H_{3}W_{\gamma }\right| \end{array}\right.$$

(73) $$\left\{ \begin{array}{c} W_{1}=W_{\alpha }-K_{1}U_{\alpha }\\ W_{2}=W_{\beta }-K_{2}U_{\beta }\\ W_{3}=W_{\gamma }-K_{3}U_{\gamma } \end{array}\right.$$

(74) $$W\left(t+1\right)=\frac{1}{3}\left(W_{1}+W_{2}+W_{3}\right)$$

where $W_{\alpha },W_{\beta }\text{,}$ and $W_{\gamma }$ specify the gray wolf location. $H_{1}$, $H_{2}$, $H_{3}$, $K_{1}$, $K_{2}$, and $K_{3}$ are scale factors.

8) The values of α,H and K are updated. If the conditions are not satisfied, go to step 2 again.
9) The output parameter 'c' and 'g' are used to construct the SVC prototype for the sake of recognition, identification and classification.

### 6.2 GOA based sparse autoencoder

Once the dimensionality of the respiratory sound data is reduced, GOA is utilized to select the essential features and it is fed to the sparse autoencoder. One of the famous metaheuristic algorithms inspired by nature is GOA [72]. The behavior of the grasshopper is mimicked and simulated here in this algorithm. Their specific group movements towards the available food sources in nature are covered by GOA. The mathematical model of GOA is quite useful to solve an optimization issue as it has the ability to imitate grasshopper's behavior in nature. GOA has acquired a reputation of deciphering real problems even with unknown space. To simulate the grasshopper's behavior, the mathematical model designed is as follows:

(75) $$P_{i}=S_{i}+G_{i}+F_{i}$$

where the position orientation of the grasshopper number i is expressed as $P_{i}$. The social interaction or the interlinkage relationship is expressed as $S_{i}$. The gravitational force is expressed as $G_{i}$. The wind flow rate is expressed as $F_{i}$. In order to represent a behavior which is random in nature, the equation is written as follows.

(76) $$P_{i}=r_{1}S_{i}+r_{2}G_{i}+r_{3}F_{i}$$

where the random numbers are denoted as $r_{`},r_{2},r_{3}$ and are present in the span of [0,1]. The distance between the grasshoppers i and j is denoted as $d_{ij}$ and is computed as $d_{ij}=\left|P_{i}-P_{j}\right|$.

The social interaction is presented as the following equation:

(77) $$S_{i}=\sum_{\begin{array}{l} j=1\\ j\neq i \end{array}}^{N}s\left(d_{ij}\right){\hat{d}}_{ij}$$

where the calculation of $G_{i}$ is done as $G_{i}=-g{\hat{e}}_{g}$. The unit vector that inclines to the center of the earth is expressed as ${\hat{e}}_{g}$. The $F_{i}$ is denoted by the equation below, where the fixed drift is denoted by u and the wind flow unit vector is denoted by ${\hat{e}}_{w}$.

(78) $$F_{i}=u{\hat{e}}_{w}$$

As per the equations presented, to simulate the grasshopper's optimization, the mathematical model used is as follows:

(79) $$P_{i}=\sum_{\begin{array}{l} j=1\\ j\neq i \end{array}}^{N}s\left(\left|P_{j}-P_{i}\right|\right)\frac{P_{j}-P_{i}}{d_{ij}}-g{\hat{e}}_{g}+u{\hat{e}}_{w}$$

A modified version of this equation is expressed so that the optimization problem can be solved.

(80) $$P_{i}^{d}=c\left[\sum_{\begin{array}{l} j=1\\ j\neq i \end{array}}^{N}c\frac{u_{bd}-l_{bd}}{2}s\left(\left|p_{j}^{d}-p_{i}^{d}\right|\right)\frac{p_{j}-p_{i}}{d_{ij}}\right]+{\hat{T}}_{d}$$

where $u_{bd}$ specifies the upper bound, $l_{bd}$ indicates the lower bound, c and $T_{d}$ represents the constant value and the time factor respectively. Once the optimization problem is solved, the features selected are fed to the sparse autoencoder.

To safeguard the quintessence of the input data in the best feasible manner and to eliminate the potential noise, an autoencoder is used as it is basically a network which includes one input, one hidden and one output layer [73]. The output data is usually simplified and the vital details from the input data are preserved which is highly useful for classification. The processing of the entire data is split into encoding and decoding junctures. The dimension of the input data is mitigated in one layer in the encoding phase. Once the decoded data appears at the hidden layer, the dimensions of the input data outstretch the similar number of neurons predetermined for their layer. For the hidden layer h, the encoding function is expressed as:

(81) $$h=encoder\left(p\right)=f\left(W_{k}\times p+b_{k}\right)$$

where $W_{k}\in R^{m\times n}$ specifies the weight matrix intermingling the input layer and the next layer. The output function is specified by f and the bias vector is mentioned by $b_{k}$. RELU is the output activation function used here. The decoding function is expressed as:

(82) $$q=decoder\left(p\right)=g\left(W_{k}\times p+b_{k}\right)$$

where $W_{k}\in R^{n\times m}$.

The training of the model is done after the encoding and decoding phases and by means of minimizing the cost function, the parameters are obtained easily and mathematically expressed as follows:

(83) $$\min\sum \left|E\left(p^{x},q^{x}\right)\right|$$

where the output data is indicated as $q^{x}$ and the original input data is specified as $p^{x}$. The reconstruction of the output values is done when the training of network is done. The updation of the parameters of the model is done as follows:

(84) $$W_{k}=W_{k}-\eta \frac{\partial E\left(p^{x},q^{x}\right)}{\partial W_{k}}$$

(85) $$b_{k}=b_{k}-\eta \frac{\partial E\left(p^{x},q^{x}\right)}{\partial b_{k}}$$

where the learning rate of the network is expressed as η. The error in SAE is denoted by E. A sparse impediment is added on the hidden representations so that the generalization or the inference of the network is increased and the training coherence of the proposed substructure of the work is enhanced. The activation of neurons is suppressed with the help of this sparse constraint in the hidden layer and so the most applicable features are easily extricated by the autoencoder. The description of the cost function in sparse autoencoder is expressed as:

(86) $$J_{sparse}\left(W,b\right)=J\left(W,b\right)+\beta \sum_{y=1}^{m}KL\left(\rho \|{\hat{\rho }}_{y}\right)$$

where the average activation of the hidden unit y is expressed as ${\hat{\rho }}_{y}$. The sparsity penalty term has a particular weight and it is expressed by β and the sparsity kernel is expressed by ρ. The sparsity of neurons is ensured in the hidden layer by Kullback-Leibler (KL) divergence. KL is expressed as:

(87) $$KL\left(\rho \|{\hat{\rho }}_{y}\right)=\rho \;\log\frac{\rho }{{\hat{\rho }}_{y}}+\left(1-\rho \right)\frac{1-\rho }{1-{\hat{\rho }}_{y}}$$

(88) $${\hat{\rho }}_{y}=\frac{1}{m}\sum_{x=1}^{m}f_{y}\left(p^{x}\right)$$

where the number of samples at unite y in the hidden layer is expressed as m. The activation of hidden neuron y is expressed as $f_{y}$.

## 7 Results and discussion

A publicly accessible ICBHI 2017 Respiratory Sound Database has been utilized in this work. Two research groups in two different countries (Portugal and Greece) collected the sound samples independently over several years in this dataset and the recording was done using three various electronic stethoscopes such as Meditron, LittC2SE, Litt3200. With the help of specialists, the labelling of the respiratory cycles was done by means of dividing them into 8 different types of lung conditions (Healthy, URTI, LRTI, COPD, Pneumonia, Bronchiectasis, Asthma and Bronchiolitis). The recording of the audio was done from the trachea and other locations in the chest. The recordings were collected from adult participants with different ages in both the clinical and non-clinical background. There are about 920 audio samples from 126 subjects in this ICBHI database. The annotation of every respiratory cycle in the dataset is done amidst 4 classes. Two broad groups are covered by this type of annotation, such as healthy and non-healthy. The division of the non-healthy category yields into wheeze and crackle and in some respiratory cycles both these issues are present. The standard metrics utilized here for the analysis was Sensitivity, Specificity and Accuracy and is represented by the following formulae as follows:

(89) $$Sensitivity=\frac{TP}{TP+FN}$$

(90) $$Specificity=\frac{TN}{TN+FP}$$

(91) $$Accuracy=\frac{TP+TN}{TP+TN+FP+FN}$$

where TP indicates True Positive, TN indicates True Negative, FP specifies False Positive and FN specifies False Negative respectively. A 10-fold cross-validation scheme has been utilized in our experiment to obtain the results for the proposed works. In the experimental analysis, wherever SVM was used, a Gaussian kernel was employed in the SVM. The Kernel and the penalty parameters are usually set in the range of $\left\{10^{-3},10^{-2},...,10^{2},10^{3}\right\}$ and the value of the regularization parameter was set at 10. In the proposed SEEMD algorithm, the noise standard deviation was set as 0.4 and the maximum number of iterations allowed in our experiment is 1000 for easy computation's sake. In the proposed RR-SRC algorithm the subspace dimension was set as 50 and the threshold parameter was chosen as 0.6 after the best results were obtained through several trial-and-error experimentation process. The number of atoms utilized in each class while dealing with K-SVD aspect of RR-SRC algorithm is {10, 20, 30, 40, 50}.

As far the DM based VMD-ELM is concerned, the hyperparameter settings are as follows. The penalty factor in the VMD is set as 300. The tolerance of the convergence criterion is set as 0.001 and time-step of the dual ascent was set as 0.5 in our experiment. All the values were uniformly initialized starting from one in our experiment. The number of hidden neurons considered in our experiment were 30 and the transfer function utilized was sigmoid. As far as the backpropagation part in ELM is concerned, the number of hidden neurons is set as 30 and the number of hidden layers is set as 1. The learning rate is assigned as 0.02 and the error target value was assigned as 0.000001.

As far as the proposed HHO-SFPDE algorithm is concerned, the population size of the HHO is set as 50 and the maximum number of iterations of HHO is allowed at 500 iterations so that the experimentation can be thoroughly run. The population size of the SFPDE is set as 75 and the maximum number of iterations of SFPDE is allowed at 500 again for a through computation. The selection of the population size is purely a heuristic choice in our experiment. The number of fitness evaluation must be more accurate and so a slightly higher population size is set to the SFPDE algorithm.

For the utilized Gray-wolf optimization, the input weights are chosen in the range of [−1,1] and the bias value is set as 0.5. The output weight matrix is dependent on the position of the gray wolf and it is initiated stochastically and generated within the range of [−1,1] respectively. The number of search agents are set as 50 in our experiment when utilizing the GWO. As far as the parameters of GOA is concerned, the grasshopper agents are chosen as 60 in our experiment and the number of iterations is set to 400. For the sparse autoencoder, the epoch size is set as 200, batch size is set as 64 and the learning rate is set as 0.04 respectively.

Table 1 shows the results of the proposed Ensemble GSSR techniques with different classifiers. A high classification accuracy of 85.39% is obtained for 2-class classification, 84.22% is obtained for 3-class classification and 82.09% is obtained for 4-class classification. Table 2 shows the results of the proposed Local information-based RR-SRC classifiers, where a classification accuracy of 86.42% is obtained for 2-class classification, 85.43% is obtained for 3-class classification and 83.23% is obtained for 4-class classification. The PCA based RR-SRC too gave a similar kind of performance where a classification accuracy of 84.64% is obtained for 2-class classification, 85.34% is obtained for 3-class classification and 83.19% is obtained for 4-class classification. Table 3 shows the results of the proposed DM based VMD-ELM classifiers and a high classification accuracy of 95.39% is obtained for 2-class classification with Manhattan distance based VMD-ELM, 90.61% is obtained for 3-class classification with Manhattan distance based VMD-ELM, and 89.27% is obtained for 4-class classification with Manhattan distance based VMD-ELM. Table 4 shows the results of the proposed HHO-SFPDE model with classifiers for a 2-class classification and a high classification accuracy of 74.67% is obtained when HHO is classified with KNN, 89.35% is obtained when SFPDE model is classified with SVM and 91% is obtained when HHO-SPFDE model is classified with SVM. Table 5 shows the results of the proposed HHO-SFPDE model with classifiers for a 3-class classification and a high classification accuracy of 72.32% is obtained when HHO is classified with RF, 87.42% is obtained when SFPDE model is classified with SVM and 90.09% is obtained when HHO-SFPDE model is classified with SVM. Table 6 shows the results of the proposed HHO-SFPDE model with classifiers for a 4-class classification and a high classification accuracy of 71.28% is obtained when HHO is classified with RF, 85.26% is obtained when SFPDE model is classified with SVM and 88.73% is obtained when HHO-SFPDE model is classified with SVM. Table 7 shows the results of the 2-class classification technique with MDS dimensionality reduction technique and SVC classifier and a high classification accuracy of 70.92% is obtained, the LLC dimensionality reduction technique with GWO-SVC classifier reported an accuracy of 85.01% and LLC dimensionality reduction technique with GOA-SA technique reported an accuracy of 86.42%. Table 8 shows the results of the 3-class classification technique with LTSA dimensionality reduction technique with SVC classifier and a high classification accuracy of 70.29% is obtained, the LLC dimensionality reduction technique with GWO-SVC classifier reported an accuracy of 82.42% and LLC dimensionality reduction technique with GOA-SA classifier reported an accuracy of 84.38%. Table 9 shows the results of the 4-class classification technique with Isomap dimensionality reduction technique and SVC classifier and a high classification accuracy of 63.13% is obtained, the LTSA dimensionality reduction technique with GWO-SVC classifier reported an accuracy of 78.3% and LTSA dimensionality reduction technique with GOA-SA technique reported an accuracy of 82.78%.

**Table 1:** Proposed Ensemble GSSR techniques.

Classifiers	2-class classification			3-class classification			4-class classification		
Classifiers	Sensitivity	Specificity	Accuracy	Sensitivity	Specificity	Accuracy	Sensitivity	Specificity	Accuracy
KNN	84.34	83.11	83.72	83.09	82.34	82.71	81.34	80.76	81.05
Adaboost	84.56	86.23	**85.39**	82.78	85.67	**84.22**	80.67	83.57	82.12
DT	83.78	82.46	83.12	80.11	81.89	81	79.12	79.78	79.45
RF	84.87	83.77	84.32	83.25	83.34	83.29	82.36	81.82	**82.09**
NBC	81.65	79.64	80.64	79.67	75.78	77.72	78.99	74.21	76.6
SVM	84.12	85.32	84.72	82.92	83.12	83.02	80.12	82.34	81.23

**Table 2:** Proposed RR-SRC

Classifiers	2-class classification			3-class classification			4-class classification		
Classifiers	Sensitivity	Specificity	Accuracy	Sensitivity	Specificity	Accuracy	Sensitivity	Specificity	Accuracy
SRC	85.78	84.46	85.12	85.45	83.11	84.28	82.02	82.22	82.12
Local Information based RR-SRC	85.64	87.21	**86.42**	84.66	86.21	**85.43**	82.34	84.12	**83.23**
PCA based RR-SRC	85.19	84.09	84.64	85.91	84.78	85.345	83.67	82.71	83.19

**Table 3:** Proposed DM based VMD-ELM.

Classifiers	2-class classification			3-class classification			4-class classification		
Classifiers	Sensitivity	Specificity	Accuracy	Sensitivity	Specificity	Accuracy	Sensitivity	Specificity	Accuracy
Euclidean distance based VMD-ELM	92.34	93.12	92.73	89.22	88.36	88.79	90.01	88.21	89.11
Manhattan distance based VMD-ELM	94.45	96.34	**95.39**	91.01	90.21	**90.61**	90.22	88.33	**89.27**
Chebyshev distance based VMD-ELM	93.67	92.67	93.17	89.34	88.76	89.05	89.37	88.47	88.92
Jaccard Index based VMD-ELM	93.81	92.87	93.34	88.56	88.39	88.47	89.66	88.03	88.84
Sorensen-Dice Index based VMD-ELM	91.22	92.21	91.715	89.71	88.71	89.21	88.41	90.11	89.26

**Table 4:** Proposed HHO-SFPDE with classifiers – 2 class classification.

Classifiers	HHO			Proposed SFPDE			Proposed HHO-SFPDE		
Classifiers	Sensitivity	Specificity	Accuracy	Sensitivity	Specificity	Accuracy	Sensitivity	Specificity	Accuracy
KNN	76.33	73.02	**74.67**	78.22	79.09	78.65	85.02	89.09	87.05
Adaboost	74.45	72.33	73.39	82.34	81.87	82.10	85.33	87.87	86.6
DT	72.78	71.45	72.11	83.67	84.23	83.95	86.45	85.67	86.06
RF	74.89	72.78	73.83	84.82	85.46	85.14	88.78	87.11	87.94
NBC	71.76	69.91	70.83	84.31	86.78	85.54	89.61	88.25	88.93
SVM	71.12	70.16	70.64	89.89	88.81	**89.35**	91.33	90.67	**91**

**Table 5:** Proposed HHO-SFPDE with classifiers – 3 class classification.

Classifiers	HHO			Proposed SFDE			Proposed HHO-SFPDE		
Classifiers	Sensitivity	Specificity	Accuracy	Sensitivity	Specificity	Accuracy	Sensitivity	Specificity	Accuracy
KNN	72.01	71.21	71.61	75.02	78.32	76.67	81.02	87.21	84.11
Adaboost	72.34	70.56	71.45	80.76	79.87	80.31	84.34	85.89	85.11
DT	70.55	70.78	70.66	82.69	83.65	83.17	85.76	83.87	84.81
RF	72.67	71.98	**72.32**	83.87	85.21	84.54	89.51	85.32	87.41
NBC	70.89	69.31	70.1	85.61	83.33	84.47	89.44	87.76	88.6
SVM	70.11	69.70	69.905	87.02	87.82	**87.42**	90.98	89.21	**90.09**

**Table 6:** Proposed HHO-SFPDE with classifiers – 4 class classification.

Classifiers	HHO			Proposed SFDE			Proposed HHO-SFPDE		
Classifiers	Sensitivity	Specificity	Accuracy	Sensitivity	Specificity	Accuracy	Sensitivity	Specificity	Accuracy
KNN	71.01	70.01	70.51	73.22	75.22	74.22	80.02	85.11	82.56
Adaboost	70.22	68.23	69.22	79.12	78.25	78.68	83.33	84.23	83.78
DT	68.34	69.66	69	80.34	81.09	80.71	84.45	81.67	83.06
RF	71.67	70.89	**71.28**	82.78	83.87	83.32	86.78	84.89	85.83
NBC	69.89	68.21	69.05	84.92	81.58	83.25	87.91	86.92	87.41
SVM	69.32	67.03	68.175	85.31	85.22	**85.26**	89.23	88.23	**88.73**

**Table 7:** Proposed GWO-SVC and GOA-SA with dimensionality reduction techniques - 2 class classification.

	SVC			GWO-SVC			GOA-SA		
	Sensitivity	Specificity	Accuracy	Sensitivity	Specificity	Accuracy	Sensitivity	Specificity	Accuracy
MDS	70.34	71.50	**70.92**	80.11	81.11	80.61	81.02	82.11	81.56
Isomap	70.23	70.86	70.54	84.37	84.27	84.32	83.33	85.23	84.28
LLE	68.78	69.79	69.28	82.86	83.87	83.36	84.46	85.58	85.02
LTSA	70.03	71.21	70.62	83.34	84.32	83.83	84.89	85.92	85.40
LLC	71.49	69.22	70.35	85.58	84.44	**85.01**	86.51	86.33	**86.42**

**Table 8:** Proposed GWO-SVC and GOA-SA with dimensionality reduction techniques - 3 class classification.

	SVC			GWO-SVC			GOA-SA		
	Sensitivity	Specificity	Accuracy	Sensitivity	Specificity	Accuracy	Sensitivity	Specificity	Accuracy
MDS	69.22	70.33	69.77	79.22	80.02	79.62	80.23	80.01	80.12
Isomap	69.46	68.45	68.95	82.34	82.33	82.33	82.90	82.22	82.56
LLE	66.78	63.89	65.33	80.68	82.47	81.57	83.87	84.34	84.10
LTSA	69.87	70.71	**70.29**	81.71	83.89	82.8	83.62	82.78	83.2
LLC	70.12	67.33	68.725	82.23	82.61	**82.42**	84.11	84.65	**84.38**

**Table 9:** Proposed GWO-SVC and GOA-SA with dimensionality reduction techniques - 4 class classification.

	SVC			GWO-SVC			GOA-SA		
	Sensitivity	Specificity	Accuracy	Sensitivity	Specificity	Accuracy	Sensitivity	Specificity	Accuracy
MDS	62.22	61.89	62.055	73.11	78.02	75.56	78.21	79.21	78.71
Isomap	63.34	62.92	**63.13**	78.23	77.33	77.78	78.23	80.22	79.22
LLE	60.67	64.34	62.505	76.67	77.46	77.06	80.56	81.36	80.96
LTSA	61.12	60.58	60.85	77.82	78.78	**78.3**	81.78	83.78	**82.78**
LLC	60.79	62.91	61.85	76.34	77.91	77.12	82.90	81.91	82.40

**Fig. 10:**
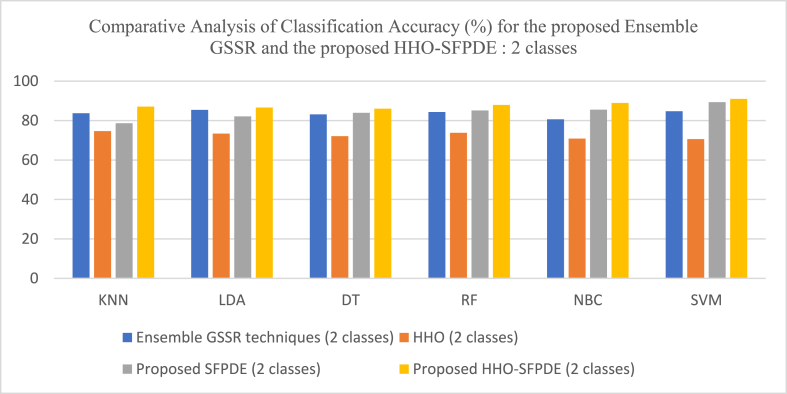
Comparative Analysis of Classification Accuracy (%) for the proposed Ensemble GSSR and the proposed HHO-SPDE (2 classes).

**Fig. 11:**
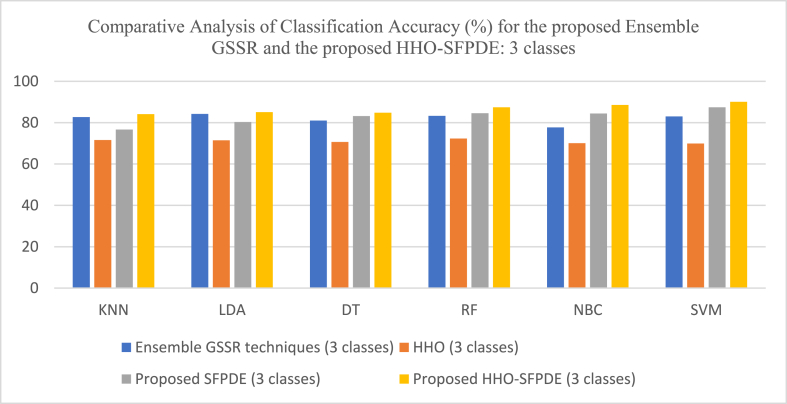
Comparative Analysis of Classification Accuracy (%) for the proposed Ensemble GSSR and the proposed HHO-SPDE (3 classes).

**Fig. 12:**
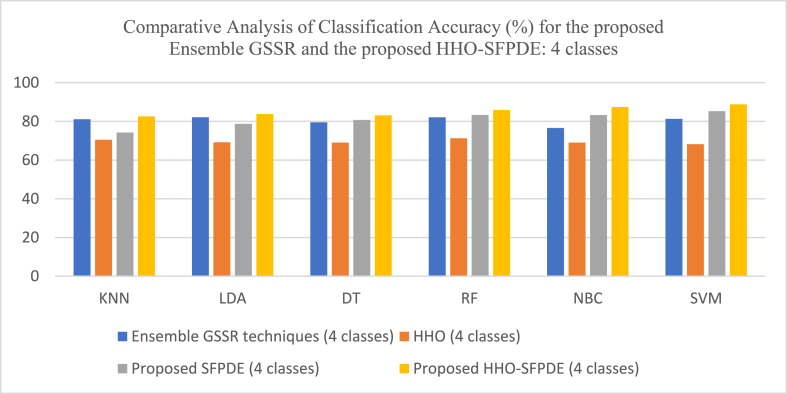
Comparative Analysis of Classification Accuracy (%) for the proposed Ensemble GSSR and the proposed HHO-SPDE (4 classes).

**Fig. 13:**
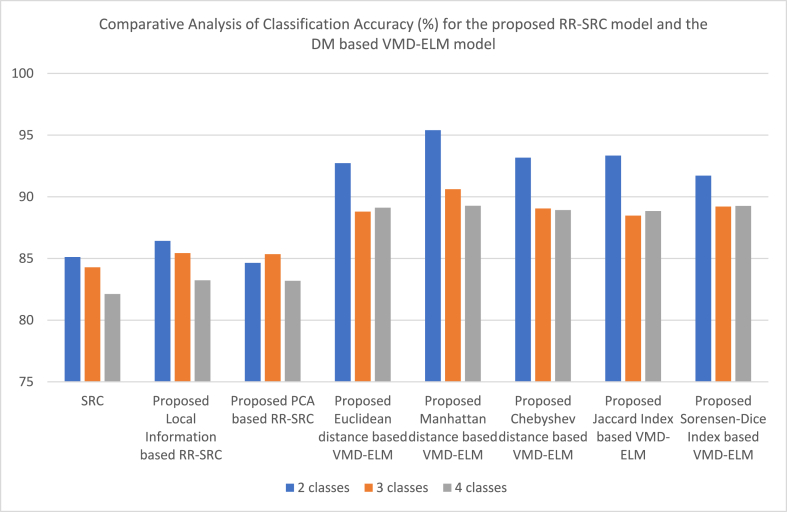
Comparative Analysis of Classification Accuracy (%) for the proposed RR-SRC model and the DM based VMD-ELM model.

**Fig. 14:**
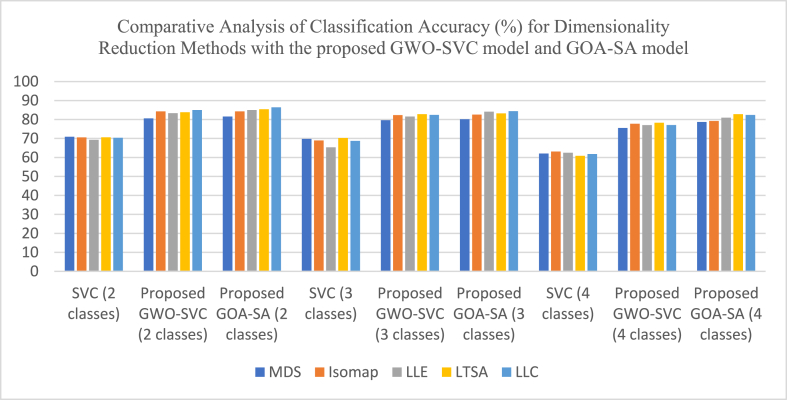
Comparative Analysis of Classification Accuracy (%) for the Dimensionality Reduction Methods with the proposed GWO-SVC model and GOA-SA model.

Fig. 10 shows the comparative analysis of Classification Accuracy (%) for the proposed Ensemble GSSR and the proposed HHO-SPDE (2 classes). Fig. 11 shows the comparative analysis of Classification Accuracy (%) for the proposed Ensemble GSSR and the proposed HHO-SPDE (3 classes). Fig. 12 shows the comparative analysis of Classification Accuracy (%) for the proposed Ensemble GSSR and the proposed HHO-SPDE (4 classes). Fig. 13 shows the comparative analysis of Classification Accuracy (%) for the proposed RR-SRC model and the DM based VMD-ELM model. Fig. 14 shows the comparative analysis of Classification Accuracy (%) for the Dimensionality Reduction Methods with the proposed GWO-SVC model and GOA-SA model.

On observing Fig. 10, it is evident that the proposed HHO-SFPDE with SVM gives us a higher classification accuracy when comparing the other models for a 2 class-classification. It is also inferred that the proposed HHO-SFPDE performs better than the ensemble GSSR model for a 3-class classification and 4-class classification from observing Fig. 11, Fig. 12. A high classification accuracy is obtained from the proposed Manhattan distance based VMD-ELM model from observing Fig. 13 and high classification accuracies are obtained from the proposed GWO-SVC and the proposed GOA-SA models when compared to the SVC model by observing Fig. 14.

### 7.1 Comparison of the proposed results with previous works done for ICBHI dataset

The comparison of the proposed results with the previous work done for ICBHI dataset is reported in Table 10.

**Table 10:** Performance comparison of the proposed works with the previous works done for ICBHI dataset.

Author and Year	Technique Used	Number of Classes	Results (%)
Minami et al. (2019)	CNN	4	Accuracy = 50%
Ma et al. (2019)	Bi-ResNet deep learning	4	Accuracy = 52.79%
Monaco et al. (2020)	DNN	2	Accuracy = 82%
Gairola et al. (2020)	ResNet 34	2	F score = 77%
Nguyen & Pernkopf 2020)	CNN	2	Accuracy = 83.7%
Shuvo et al. (2020)	Lightweight CNN	3	Accuracy = 98.9%
Naqvi et al. 2020)	ML classifiers	3	Accuracy = 97.7%
Yang et al. (2020)	STFT + wavelet features + Bi-ResNet	4	Accuracy = 50.16%
Ngo et al. (2020)	CNN with Autoencoders	4	Accuracy = 49%
Pham et al. (2020)	Inception NN	4	Accuracy = 86%
Nguyen & Pernkopf (2020)	CNN	4	Accuracy = 78.4%
Acharya & Basu (2020)	Hybrid CNN-RNN model	4	Accuracy = 71.81%
Mukherjee et al. (2021)	MLP	2	Accuracy = 99.2%
Ntalampiras & Potamitits (2021)	DAG-HMM	4	Accuracy = 50.1%
Borwankar et al. (2022)	Multilayer CNN	3	F score = 0.99
Tasar et al. (2022)	KNN	3	Accuracy = 99.31%
Petmezas et al. (2022)	CNN-LSTM with Focal Loss	4	Accuracy = 76.39%
**Proposed Works- Set 1**	Ensemble GSSR with Adaboost	2	Accuracy = 85.39%
	RR-SRC	2	Accuracy = 86.42%
	**Manhattan VMD-ELM**	**2**	**Accuracy** = **95.39%**
	HHO-SFPDE with SVM	2	Accuracy = 91%
	GWO-SVC	2	Accuracy = 85.01%
	GOA-SA	2	Accuracy = 86.42%
**Proposed Works- Set 2**	Ensemble GSSR with Adaboost	3	Accuracy = 84.22%
	RR-SRC	3	Accuracy = 85.43%
	**Manhattan VMD-ELM**	**3**	**Accuracy** = **90.61%**
	HHO-SFPDE with SVM	3	Accuracy = 90.09%
	GWO-SVC	3	Accuracy = 82.42%
	GOA-SA	3	Accuracy = 84.38%
**Proposed Works- Set 3**	Ensemble GSSR with Adaboost	4	Accuracy = 82.09%
	RR-SRC	4	Accuracy = 83.23%
	**Manhattan VMD-ELM**	**4**	**Accuracy** = **89.27%**
	HHO-SFPDE with SVM	4	Accuracy = 88.73%
	GWO-SVC	4	Accuracy = 78.3%
	GOA-SA	4	Accuracy = 82.78%

On the analysis of Table 10, the proposed works gave good results when compared to the previous works. For a 2-class classification, the proposed ensemble GSSR with Adaboost technique reported an accuracy of 85.39%, RR-SRC reported an accuracy of 86.42%, Manhattan VMD-ELM reported an accuracy of 95.39%, HHO-SFPDE with SVM reported an accuracy of 91%, GWO-SVC reported an accuracy of 85.01% and GOA-SA reported an accuracy of 86.42%. For a 3-class classification, the proposed ensemble GSSR with Adaboost technique reported an accuracy of 84.22%, RR-SRC reported an accuracy of 85.43%, Manhattan VMD-ELM reported an accuracy of 90.61%, HHO-SFPDE with SVM reported an accuracy of 90.09%, GWO-SVC reported an accuracy of 82.42% and GOA-SA reported an accuracy of 84.38%. For a 4-class classification, the proposed ensemble GSSR with Adaboost technique reported an accuracy of 82.09%, RR-SRC reported an accuracy of 83.23%, Manhattan VMD-ELM reported an accuracy of 89.27%, HHO-SFPDE with SVM reported an accuracy of 88.73%, GWO-SVC reported an accuracy of 78.3% and GOA-SA reported an accuracy of 82.78%.

As far as statistical tests are concerned, when implementing Cohen's Kappa coefficient for the features obtained, the values obtained were in the range of 0.8–1, proving that it reaches a very good agreement amongst the features. There were distinct and clear variations in the values of the features obtained when implementing Friedman test analysis. When the Kruskal Wallis test was implemented, the value obtained was less than 0.01 and when the 2-sided Wilcoxon rank sum test was implemented, the values obtained were less than 0.05 thereby a high confidence level is achieved. As far as the computational complexity was considered the following was obtained. For Ensemble GSSR with Adaboost, RR-SRC model and Manhattan VMD-ELM model, a complexity of $O\left(n^{3}\;\log\;n\right)$ was obtained. For HHO-SPFDE with SVM, a complexity of $O\left(n^{4}\;\log\;2\;n\right)$ was obtained and for GWO-SVC and GOA-SA, a complexity of $O\left(n^{4}\;\log\;n\right)$ was obtained.

As far as the limitations of the study are concerned, the correct combinations of the techniques must be utilized to get the correct results, otherwise it would result in poor results. More when using metaheuristic algorithms-based classification, the correct tuning of parameters should be provided while simulation otherwise it would result in poor results. Moreover, a very high time operating probability is not present in the work which will be addressed in the future works. The current works cannot be utilized for telemedicine applications as of now as more concepts incorporating wireless theory and communication systems must be incorporated in the work.

## 8 Conclusion and future works

For the examination of patients with respiratory disorders, a widely used technique is auscultation. One of the most important medical devices available is stethoscope as it is non-invasive, informative, and available in real-time. For analyzing respiratory diseases, it is highly useful and good information on different pathological conditions of lungs is provided by the abnormal respiratory sounds. In this work, five hybrid interpretable strategies with ensemble techniques have been applied and the analyses concludes that a highest classification accuracy of 95.39% has been obtained for a 2-class classification when Manhattan VMD-ELM is implemented, a high classification accuracy of 90.61% has been obtained for a 3-class classification when Manhattan VMD-ELM is implemented and a high classification accuracy of 89.27% has been obtained for a 4-class classification when Manhattan VMD-ELM is implemented. The proposed methods have surpassed a lot of previous results and can be applied to other signal processing techniques too. Also, the proposed methods have a high real time operating probability as an in-depth mathematical model has been provided for all of them. Future works aim to propose more hybrid models with ensemble techniques so that the respiratory sound classification can be more accurately classified. Also, an automated respiratory sound classification system for telemedicine applications is considered so that the remote treatment could be made feasible and accessible throughout the world for the betterment of the human community.

## Author contribution statement

SUNIL KUMAR PRABHAKAR: Conceived and designed the experiments; Performed the experiments; Analyzed and interpreted the data; Contributed reagents, materials, analysis tools or data; Wrote the paper.

DONG-OK WON: Performed the experiments; Contributed reagents, materials, analysis tools or data; Wrote the paper.

## Data availability statement

Data associated with this study has been deposited at https://bhichallenge.med.auth.gr/ICBHI_2017_Challenge.

## Additional information

No additional information is available for this paper.

## Declaration of competing interest

The authors declare that they have no known competing financial interests or personal relationships that could have appeared to influence the work reported in this paper.
